# Studying the interaction between PEX5 and its full-length cargo proteins in living cells by a novel Försteŕs resonance energy transfer-based competition assay

**DOI:** 10.3389/fcell.2022.1026388

**Published:** 2022-11-03

**Authors:** Bernhard Hochreiter, Hugo Malagon-Vina, Johannes A. Schmid, Johannes Berger, Markus Kunze

**Affiliations:** ^1^ Institute for Vascular Biology and Thrombosis Research, Center for Physiology and Pharmacology, Medical University of Vienna, Vienna, Austria; ^2^ Department of Cognitive Neurobiology, Center for Brain Research, Medical University of Vienna, Vienna, Austria; ^3^ Department of Pathobiology of the Nervous System, Center for Brain Research, Medical University of Vienna, Vienna, Austria

**Keywords:** peroxisomes, PEX5, PTS1, PEX14, FRET, fitting algorithm, competition experiments

## Abstract

The import of the majority of soluble peroxisomal proteins is initiated by the interaction between type-1 peroxisomal targeting signals (PTS1) and their receptor PEX5. PTS1 motifs reside at the extreme C-terminus of proteins and consist of a characteristic tripeptide and a modulatory upstream region. Various PTS1-PEX5 interactions have been studied by biophysical methods using isolated proteins or in heterologous systems such as two-hybrid assays, but a recently established approach based on Försters resonance energy transfer (FRET) allows a quantifying investigation in living cells. FRET is the radiation-free energy transfer between two fluorophores in close proximity and can be used to estimate the fraction of acceptor molecules bound to a donor molecule. For PTS1-PEX5 this method relies on the measurement of FRET-efficiency between the PTS1-binding TPR-domain of PEX5 tagged with mCherry and EGFP fused to a PTS1 peptide. However, this method is less suitable for binding partners with low affinity and protein complexes involving large proteins such as the interaction between full-length PTS1-carrying cargo proteins and PEX5. To overcome this limitation, we introduce a life-cell competition assay based on the same FRET approach but including a fusion protein of Cerulean with the protein of interest as a competitor. After implementing the mathematical description of competitive binding experiments into a fitting algorithm, we demonstrate the functionality of this approach using known interaction partners, its ability to circumvent previous limitations of FRET-measurements and its ability to study the interaction between PEX5 and its full-length cargo proteins. We find that some proteins (SCP2 and AGXT) bind PEX5 with higher affinity than their PTS1-peptides alone, but other proteins (ACOX3, DAO, PerCR-SRL) bind with lower but reasonable affinity, whereas GSTK1 binds with very low affinity. This binding strength was not increased upon elongating the PEX5 TPR-domain at its N-terminus, PEX5(N-TPR), although it interacts specifically with the N-terminal domain of PEX14. Finally, we demonstrate that the latter reduces the interaction strength between PEX5(N-TPR) and PTS1 by a dose-dependent but apparently non-competitive mechanism. Altogether, this demonstrates the power of this novel FRET-based competition approach for studying cargo recognition by PEX5 and protein complexes including large proteins in general.

## 1 Introduction

Most protein transport processes are initiated by the specific interaction between a targeting signal within the proteins primary sequence and a corresponding receptor protein specifying one subcellular compartment (Kunze and Berger, 2015). Among these compartments, peroxisomes are characterized by a single membrane, the enzymatic equipment for various specific metabolic pathways including the degradation of various fatty acids or hydrogen peroxide (Waterham et al., 2016), and an import machinery for fully folded soluble proteins (Kim and Hettema, 2015). The importance of peroxisomes for human physiology is highlighted by a variety of inherited human diseases caused by a complete dysfunction of peroxisomes (ZSD, Zellweger syndrome disorder) or a defect of one of the peroxisomal enzymes (Waterham et al., 2016). Protein import of soluble proteins is induced by the interaction between peroxisomal targeting signals residing either at the extreme C-terminus (type-1 peroxisomal targeting signal, PTS1) or in close proximity to the N-terminus (type-2 peroxisomal targeting signal, PTS2) (Brocard and Hartig, 2006; Kunze, 2020). These targeting signals are recognized by the cognate soluble receptors PEX5 or PEX7 (Dahan et al., 2021), respectively, the latter acting together with a co-receptor (Kunze, 2020). These receptor proteins mediate the transport to and across the peroxisomal membrane and become recycled to the cytosol upon release of their cargo proteins into the peroxisomal lumen (Francisco et al., 2017). Most matrix proteins harbor a PTS1, which has been originally described as a C-terminal tripeptide consisting of serine-lysine-leucine (-SKL) or conserved variants thereof (Gould et al., 1989), but later-on a broader variety of tripeptides was shown to interact with the receptor and the preceding sequence also contributes to the interaction (Lametschwandtner et al., 1998; Brocard and Hartig, 2006; Ghosh and Berg, 2010; Hagen et al., 2015; Hochreiter et al., 2020). The PTS1-binding domain of PEX5 has the characteristic shape of tetratricopeptide (TPR)-proteins and interacts with the PTS1 *via* a deep cave into which PTS1 motifs are inserted (Gatto et al., 2000; Stanley et al., 2006; Burgi et al., 2021). The specificity of the interaction between PEX5 and its cargo has been initially attributed to the PTS1 motif alone (Gatto et al., 2000; Brocard and Hartig, 2006; Ghosh and Berg, 2010), but the resolution of the 3D-structure of PEX5 together with either sterol carrier protein 2 (SCP2) (Stanley et al., 2006) or alanine-glyoxylate aminotransferase (AGXT) (Fodor et al., 2012; Fodor et al., 2015) as bound cargo revealed that additional interphases exist. However, mutating individual PEX5 residues participating in this interphase had only moderate effects on the interaction strength with SCP2 (Williams et al., 2011), or import efficiency (Fodor et al., 2012; Fodor et al., 2015). The complexity of receptor-cargo interactions is further increased by the fact that many peroxisomal proteins act as dimers or oligomers, and for some of them, a piggy-back-like import mechanism of PTS1-free proteins has been demonstrated (Leon et al., 2006; Islinger et al., 2009). However, such piggy-back transport is not applicable for all peroxisomal proteins (Tanaka et al., 2008) and their general importance for peroxisomal import has been debated (Dias et al., 2016). Surprisingly, a PEX5 variant consisting of the TPR-domain together with a short N-terminal extension (N-TPR) was found to resolve human catalase tetramers in *in vitro* experiments (Freitas et al., 2011), resulting in the attribution of chaperone activity to the N-terminal part of PEX5 (Francisco et al., 2013), which might prevent proteins from complete cytosolic oligomerization. This effect was reduced by the N-terminal domain (NTD) of PEX14 bound to this N-terminal extension of PEX5 (Neufeld et al., 2009; Freitas et al., 2011; Neuhaus et al., 2014).

Various detailed studies investigated the binding strength between PEX5 from diverse species and different PTS1-peptides using isolated proteins (Gatto et al., 2003; Maynard et al., 2004; Maynard and Berg, 2007; Ghosh and Berg, 2010), yeast two-hybrid assay (Lametschwandtner et al., 1998) or more advanced techniques such as mass-spectrometry-based interaction tests (Skoulding et al., 2015; Cross et al., 2017; Rosenthal et al., 2020) and found different affinities ranging across several orders of magnitude. We recently introduced a novel application of FRET-efficiency measurements combined with a fitting algorithm to calculate numeric values for the apparent interaction strength between proteins (Hochreiter et al., 2019) and demonstrated its versatility to study the interaction between PEX5 and diverse PTS1 peptides in living cells (Chong et al., 2019; Hochreiter et al., 2020).

**FIGURE 1:**
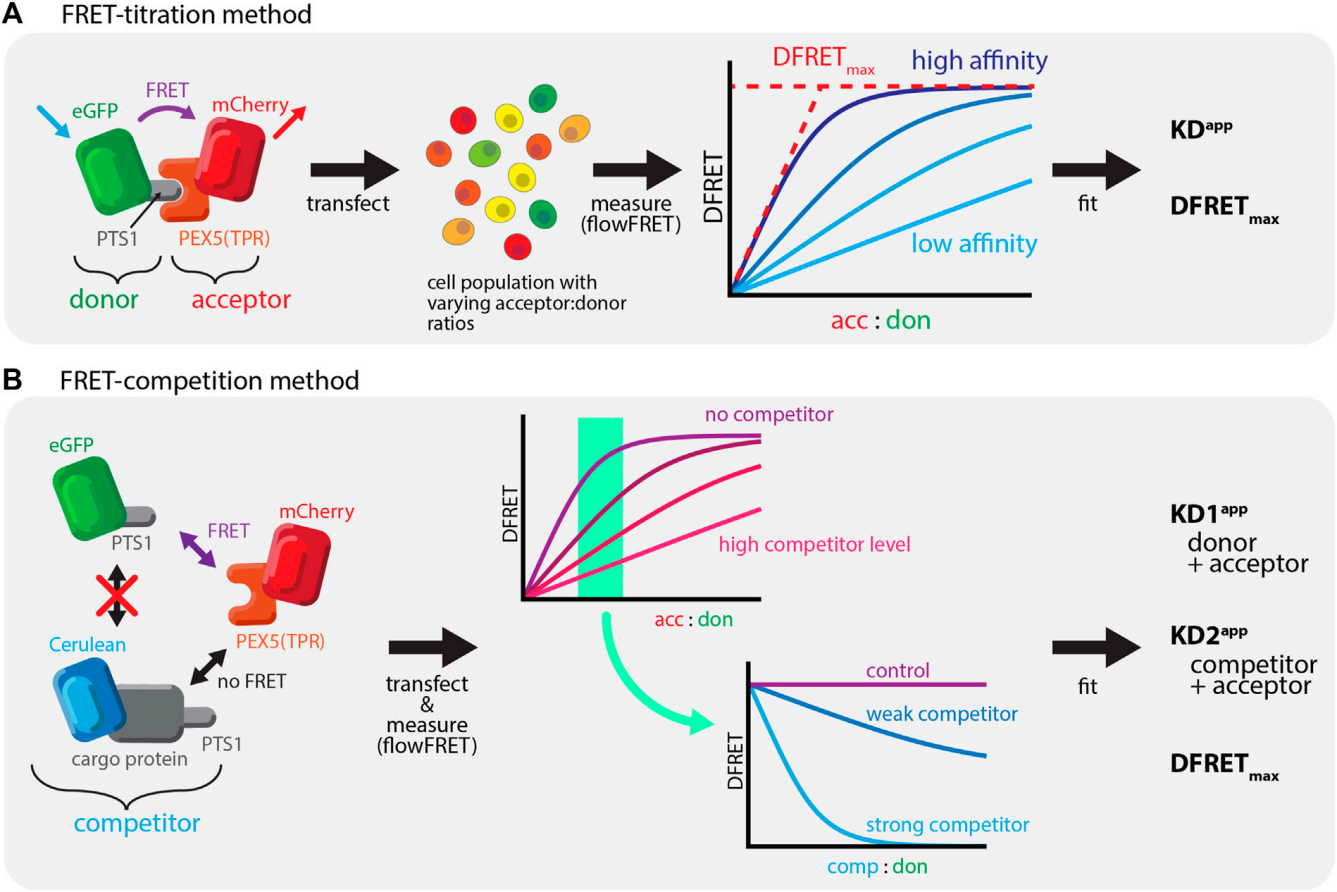
Studying PEX5-cargo interactions using FRET-measurements: **(A)** Bimolecular FRET measurements: *left*: FRET as radiation free energy transfer between fluorescent proteins in close proximity is used to measure the interaction between mCherry-extended by the TPR-domain of PEX5 (PEX5 (TPR)) and EGFP equipped with a PTS1; *middle*: a large number of cells expressing these proteins are systematically investigated using a flow-cytometer, *right*: saturation curves can be obtained by plotting the acc:don ratio against a measure of energy transfer efficiency (DFRET), in which the slope reflects the affinity and the saturation level the distance of the fluorogenic centers; **(B)** Competition based FRET experiments: *left*: when studying a defined FRET-pair (mCherry-PEX5(TPR) and EGFP-PTS1) the presence of a third protein also interacting with the acceptor (Cerulean-cargo-PTS1) exerts a competitive effect; *middle*: when plotting a saturation curve (acc:don) an increasing amount of competitor changes its shape to suggest a reduction of the interaction strength of the FRET-pair (minor slope) in a dose dependent manner; *right*: plotting DFRET against the competitor to donor ratio (comp:don) results in traditional decay curves, in which the slope of the decay reflects the relative affinity of the competitor (to the donor).

Försters resonance energy transfer (FRET) is a well-established method to investigate protein-protein interactions (PPI), which relies on the radiation-free energy transfer between a donor and an acceptor protein (Bunt and Wouters, 2017; Okamoto and Sako, 2017). The effectivity of the transfer depends on the distance between the fluorophores but can also be used to estimate the fraction of donor and acceptor molecules engaged in a complex, which is determined by the affinity of the two proteins. Thus, the quantitative determination of a normalized measure of energy transfer (donor normalized FRET, DFRET) together with an estimation of the relative abundance of donor and acceptor molecules per cell allows the estimation of key parameters. These are a measure for the apparent interaction strength (KD^app^) and the plateau level of the DFRET saturation curve (DFRETmax). However, in combination with high-throughput measurements such as flow cytometry (flowFRET) it can serve as a powerful tool to investigate PPI (Hochreiter et al., 2019) (Figure 1A). To study PTS1-PEX5 interactions, we investigated FRET-efficiency between the mCherry-tagged TPR-domain of PEX5 [mCherry-PEX5 (TPR)] and different PTS1 peptides fused to EGFP in murine embryonic fibroblasts obtained from a PEX5-deficient KO-mouse [pex5^−/−^ (Baes et al., 1997)], in which the binding partners remain in the cytosol. Certainly, the gold-standard for PEX5-cargo recognition is its interaction with full-length proteins in living cells. However, as the sensitivity of FRET efficiency measurements is limited by the distance between donor and acceptor domains and thus often by the size of the interacting proteins, we had to adapt our flowFRET method to study full-length PTS1-carrying cargo proteins.

In this manuscript we describe a FRET-based quantifying competition method, which allows the first study of PPIs between PEX5 and diverse full-length PTS1-carrying cargo proteins in living cells. We recently described qualitatively the phenomenon of competition (Hochreiter et al., 2020), which reduces the average DFRET value of populations depending on the competitor level and its affinity (Figure 1B). Moreover, we verify the interaction between PEX14(NTD) and PEX5 and demonstrate that this interaction modulates the interaction strength between PEX5 and PTS1-carrying proteins in a non-competitive manner.

## 2 Materials and methods

### 2.1 Cloning procedure

Restriction enzymes, Klenow polymerase were obtained from Fermentas/Thermo-Fisher (United States), the TA Cloning™ Kit (with pCR™2.1 Vector, without competent cells) from Thermo. PCR-reactions were performed with high-fidelity PCR (PrimeSTAR high-fidelity PCR) from Takara (JP); *in vitro* mutagenesis was performed using QuikChange® II XL Site-Directed Mutagenesis Kit from Stratagene/Agilent (US) and by independent PCR reactions using high fidelity polymerase (Pfu-I, Takara) a subsequent digestion with DpnI (Thermo-Fisher).

### 2.2 Plasmids (*cf.* Supplementary Table S3)

The EGFP-PTS1 expression plasmids were obtained by digesting EGFP-C3 (Clontech) with restriction enzymes HindIII and BglII and ligate it with the annealed oligonucleotides encoding the PTS1 of *Mm*SCP2, *Hs*AGT, *Hs*DAO, *Hs*GSTK1, *Hs*PECR, *Ss*PerCR-SRL, *MmZ*ADH2. The following expression plasmids were available [EGFP-*Mm*ZADH2 (Wiese et al., 2007), EGFP-*Hs*GSTK1 (Wang et al., 2013)] or kindly provided [EGFP-*Hs*DAO kindly provided by Loredano Pollegioni (Sacchi et al., 2011), EGFP-*Hs*AGT by Wolfgang Schliebs (Fodor et al., 2012)].

The ORF for pig PerCR-SRL was obtained by PCR using oligonucleotides Oli_2719 and Oli_2720 (*cf.* Supplementary Table S3) and the expression plasmid for PerCR-SRL (Tanaka et al., 2008) as template, the ORFs for murine SCPx was obtained by PCR using oligonucleotides Oli_901 and Oli_902 and mouse liver cDNA as template, the ORF for SCP2 was generated by PCR using oligonucleotides Oli_2615 and Oli_902 using EGFP-SCPx as template. PCR-fragments were treated with Taq-polymerase to add an additional A at the 3´-end and ligated to pCR2.1 according to manufacturer´s instructions and the correct sequence of the ORFs was confirmed by sequencing. The ORF of SCP2 or mutated variants thereof were cloned as NcoI/EcoRI-fragments and that of SCPx as XhoI/EcoRI-fragment into EGFP-C1 (Clontech) digested with the same restriction enzymes. The ORF of PerCR-SRL was cloned as BglII/SalI fragment into EGFP-C1 digested with the same restriction enzyme. The mutations in EGFP-SCP2 were either introduced by PCR using oligonucleotides (Oli_2736 and Oli_2737) including the point mutations for G139L and AKV, respectively, or by *in vitro* mutagenesis for and the double mutation E35K/K38E using oligonucleotides Oli_3002 and Oli_3003. Cerulean-tagged variants of full-length proteins were obtained by digesting the corresponding EGFP-plasmids by NheI/BsrGI and inserting the ORF of Cerulean obtained by digesting Cerulean-C1 with the same enzymes.

*mCherry-PEX5(N-TPR)* was obtained by cloning the SaclI/BamHI fragment of full-length PEX5 into mCherry-C1 digested with the same restriction enzymes and subsequently inserting the TPR-domain of PEX5 obtained from plasmid mCherry-PEX5 (TPR) (Hochreiter et al., 2020) as BamHI/BamHI fragment.

#### 2.2.1 PEX14(NTD)-EGFP

The N-terminal domain of PEX14 was obtained by PCR using oligonucleotides Oli_1529 and Oli_2799 and after subcloning into pCR2.1 the XhoI/BamHI fragment was inserted into EGFP-N2 (Clontech).

#### 2.2.2 PEX14(NTD)-cerulean

The DNA-fragment encoding the PEX14(NTD) was cloned as XhoI/BamHI-fragment into Cerulean-N1 digested with the same enzymes. Subsequently, the reading frame was shifted by first digesting the plasmid with BamHI, the 5´-overhang ends were filled up Klenow-polymerase (Thermo) and the fragment was religated.

### 2.3 FlowFRET experiments

Experiments were performed as described before (Hochreiter et al., 2019; Hochreiter et al., 2020), but a general description is summarized here: Pex5^−/−^ MEF cells were cultivated in DMEM (10%FBS). One day prior to transfection, cells were seeded in 48-well plates. The confluency of cells at the time of transfection must not exceed 70%. Cells were transfected with Turbofect™ (Thermo Scientific™ R0531). The amount of DNA used was optimized to be half the amount according to protocol (i.e., per 48-well: 0.25 µg DNA + 1 µl Turbofect in 50 µl serum free medium). To span a wide array of protein concentration ratios, multiple wells contained the same sample at different transfection ratios. The transfection mixture was incubated at room temperature for 15–20 min and pipetted onto the cells. Cells were centrifuged in a swing-out centrifuge at 300 g for 30 min and subsequently incubated overnight. Transfection efficiency was usually around 20–40% with virtually no cell death. Each experiment also contained necessary controls (i.e.,: untransfected cells; single fluorophores: EGFP, mCherry, Cerulean; fusion proteins: mCherry-EGFP, mCherry-Cerulean). 24 h past transfection, cells were washed with PBS and trypsinized in small batches of no more than eight samples at a time (multiple wells with the same DNA at different transfection ratios were often combined). Cells were immediately measured on the CytoFLEX (Beckman Coulter) flow cytometer at “fast” speed (∼1min per sample). Donor channel: ex: 488, em: 525/40; Acceptor channel: ex: 561, em: 610/20; FRET channel: ex: 488, em:610/20; Competitor channel: ex: 405, em: 450/45. Data was exported from CytExpert as fcs files and subsequently processed in R to obtain and the measured raw data and calculated values according to our previously published method (Hochreiter et al., 2019).

### 2.4 Fitting algorithm

Given the equations in (Wang, 1995), the theoretical DFRET (tDFRET) is calculated using [1–7]:

$$tDFRET=\left[AB\right]*\frac{DFRETmax}{\left[B\right]}$$ (1)

$$\left[AB\right]=\frac{\left[B\right]*\left\{2\sqrt{\left(a^{2}-3b\right)}\cos\left(\theta /3\right)-a\right\}}{3KD1+\left\{\left\{2\sqrt{\left(a^{2}-3b\right)}\cos\left(\theta /3\right)-a\right\}\right\}}$$ (2)

$$tDFRET=\frac{\left[B\right]*\left\{2\sqrt{\left(a^{2}-3b\right)}\cos\left(\theta /3\right)-a\right\}}{3KD1+\left\{\left\{2\sqrt{\left(a^{2}-3b\right)}\cos\left(\theta /3\right)-a\right\}\right\}}*\frac{DFRETmax}{\left[B\right]}$$ (3)

Where:

a=KD1+KD2+D+C−A (4)

b=KD2(D−A)+KD1(C−A)+KD1∗KD2 (5)

c=−KD1∗KD2∗A (6)

and

$$\theta =\cos^{-1}\left(\frac{-2a^{3}+9ab-27c}{2\sqrt{{\left(a^{2}-3b\right)}^{3}}}\right)$$ (7)

with

A = amount of acceptor
B = amount of donor
C = amount of competitor
KD1 = Donor affinity
KD2 = Competitor affinity
AB = amount of acc.don pair

A fitting algorithm was used to find the parameters: KD1^app^, KD2^app^ and DFRETmax, by minimizing the mean squared error (MSE) between the observed DFRET (oDFRET) and the tDFRET [3], given different parameter ranges

$$minimize:\;\frac{1}{n}\overset{n}{\underset{i=1}{\sum }}{\left(oDFRET-tDFRET\right)}^{2}$$ (8)

Because of the complexity of the equations, and the fact that the KD1 and KD2 ranges are from one to >1e12, and DFRETmax ranges are from 0.01 to >0.5, the fitting algorithm used is a modified grid search, with a dynamic range selection to avoid local minima.

For any given data set, an initial fitting was obtained by averaging the resulting fitted parameters of 3 wide searches (covering the big range of the parameters (KD1, KD2 = 1 to 1e12 and DFRETmax = 0.01–0.5) performed in randomly selected subsamples of the original data set (80% of the data). This was to avoid local minima given by the entirety of the data. Then, those averaged parameters were used as the center of new ranges (given by expanding both sides by an area predefined by the user). This created a more localized grid than the initial one. The new fitted parameters that minimized the MSE were then again assumed to be the centers of new ranges, and in iteratively manner, approach the global minimum MSE. The search was stopped when the difference between a previous MSE and a new given MSE was less than 1e-6%.

### 2.5 Statistics

Unless stated different in the text, all statistics were non-parametric. To explore differences between multiple groups, the proper ANOVA was used (1-way, 2-way or 3-way) and Tukey-Kramer post-hoc test for multi-comparisons. An overview of all statistical tests is available in Supplementary Table S1.

#### 2.5.1 Fitting results and their comparisons

An overview, the numerical results of individual fittings of the different data sets and a descriptive statistic (mean and SD) are available in Supplementary Table S2. To reflect the special combination of flowFRET measurements (primary data) and the computational extraction of key parameters of interest in each independent experiment (inference) we evaluated for complex experiments the reliability of the ranking of affinities between experiments using a bootstrapping method and for less complex experiments a direct comparison of pairs of proteins of interest defined by specific research questions using a paired t-test, but to avoid a confusion of comparing data and comparing prediction results the results of the latter are indicated only in Supplementary Table S2.

#### 2.5.2 Bootstrapping method

The *p* values for ranking of the predicted affinities among the different experiments (Figures 4D,F, 5G, 6J) were compared by bootstrapping methods. We determined the consistency in the rankings over the days for different plasmids by calculating the total sum of the absolute differences between the rankings assigned to a plasmid over different days (e.g., if ACOX3 is ranked 1, 3, and 5 over the course of 3 days, the total sum of their differences is 16). We then sum all these values for all the plasmids in the experiments. Using bootstrap methods, we calculate the probabilities of obtaining the same values of summarized differences by randomizing the original data into corresponding groups. Please note that it is not the probability of getting the same ranking orders for all the days but rather the probability of obtaining relatively similar distances between their rankings [it was done one million (1e6) times]. If over 3 days 2 plasmids have 1, 1, 1, and 2, 2, 2, their value sum is the same as 2,2,2 and 1,1,1, which means we are not testing the actual exact ranking but the probability of obtaining a similarly consistent ordination.

#### 2.5.3 Paired t-tests

For the comparison of selected pairs of proteins a paired t-test was used (*p* < 0,05), the results of which can be found in Supplementary Table S2.

#### 2.5.4 Plots and curve progressions

**FIGURE 2:**
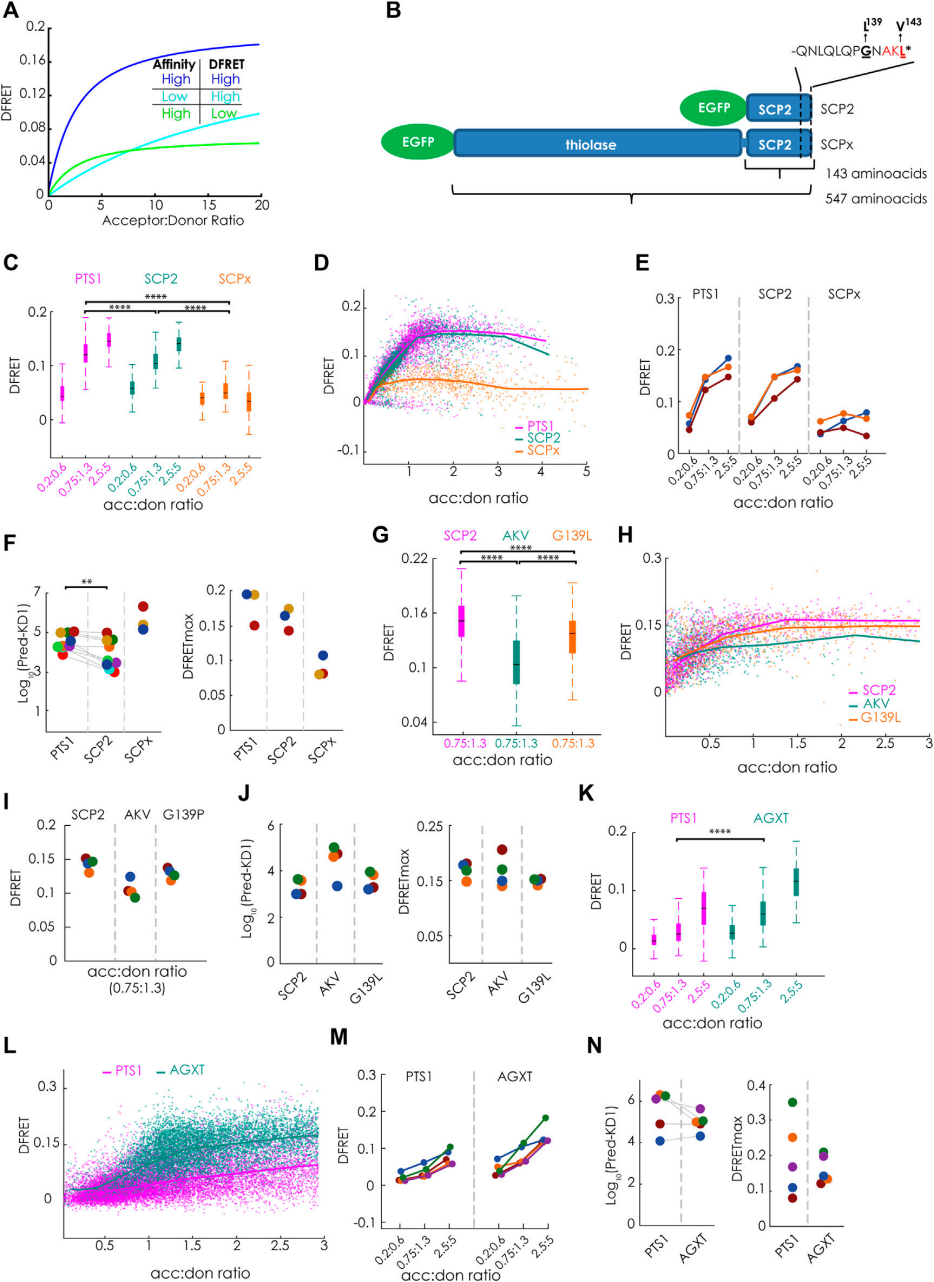
Bimolecular flowFRET analysis of protein complexes involving large proteins or low affinity binding partners: **(A)** Traditional limitations of FRET-measurements are protein complexes involving large proteins (low DFRETmax) (green) or low affinity binding partners (cyan) compared to complexes involving small proteins with high affinity (blue); **(B)** Schematic depiction of the PTS1-carrying murine proteins SCPx and SCP2 sharing the same C-terminal domain, but differing in protein size due to an additional thiolase domain in SCPx, the last 12 amino acids encoding the PTS1 (PTS1(SCP2)) are depicted separetely; **(C–N)** pex5^−/−^ MEF expressing mCherry-PEX5(TPR) as acceptor and various EGFP-tagged donor molecules were analyzed by flow cytometry to measure a large number of cells; **(C–F)** donors: EGFP-PTS1(SCP2) (12aa), EGFP-SCP2 (SCP2: 143aa) and EGFP-SCPx (SCPx: 547aa), **(C)** comparison of DFRET values of three subpopulations of cells each sharing the same acc:don ratio; **(D)** DFRET values of all cells to generate a saturation curves; **(E)** comparison of the median DFRET values of the groups described in **(C)** for three independent experiments; **(F)** results of computational extraction of the key parameter KD^app^ (left) and DFRETmax (right), each point represents one experiment, the same color indicate the same day; **(G–J)** donors: EGFP-SCP2, EGFP-SCP2(AKL→AKV) and EGFP-SCP2(G139L) data are depicted as in **(C–F)**; **(K–N)** donors: EGFP-PTS1(AGXT) and EGFP-AGXT, data are depicted are in **(C–F)**; Statistics: C,G,K: box blots with quartiles and ranges, multicomparison “Tukey-Kramer” for cells sharing acc:don 0,75 < x < 1,3 (C: PTS1 vs. SCP2 = 4.30e-14, PTS1 vs. SCPx < 1e-100, SCP2 vs. SCPx = < 1e-100; **(G)** SCP2 vs. AKV < 1e-100, SCP2 vs. G139P< = 1.445e-07, AKV vs. G139*p* = 2.0431e-15; **(I)** PTS1 vs. AGXT < 1e-100); **(F)** Wilcoxon signed rank test (*p* = 0.0039 for 9 pairs).

Axes values are depicted on each plot. Data was analysed within the ratios shown in the figures or stated in the figure legends. To plot the curve progressions (e.g., Figure 2D or Figure 4B), the median DFRET value for different ratios (acceptor to donor or competitor to donor, depending on the plot) was calculated. The ratios used were from 2^−4^ to 2^8^ in increments of 2^0.5^. The median DFRET values was then plotted in the place where the median ratio (for each interval) is located.

## 3 Results

### 3.1 Limits of the bimolecular försters resonance energy transfer-based method

FlowFRET proved to be a valuable tool for studying PEX5-PTS1 interactions, but it was unclear whether the approach was suitable for the study of full-length cargo proteins, because a large size reduces the DFRETmax (Figure 2A) and thus deteriorates the signal to noise ratio. Similarly, only low DFRET signal intensities are obtained for binding partners with weak interaction, because only a small fraction of donor is engaged with acceptor proteins limiting the power of the method as well (Figure 2A). Thus, we first studied the effect of protein size on the power of flowFRET taking advantage of two protein variants of the murine sterol-carrier protein (mSCP) sharing the C-terminal domain. These are the 143 amino acids (aa) long SCP2 protein consisting solely of the characteristic SCP-domain and the 547aa SCPx protein comprised of the SCP-domain and an additional N-terminal thiolase domain (Figure 2B). Using flowFRET experiments in pex5^−/−^ MEF, we studied the interaction between mCherry-PEX5 (TPR) and EGFP-tagged variants of these two proteins (EGFP-SCP2, EGFP-SCPx) or EGFP-PTS1 encoding the C-terminal 12 amino acids of SCP2 (EGFP-PTS1(SCP2) as described before (Hochreiter et al., 2020). As the composition of data sets markedly affects the output (Hochreiter et al., 2019) we selected subsets of cells sharing acceptor to donor ratios (acc:don) within a very narrow range and compared DFRET values as proxies for the interaction strength at three different ratios (Figure 2C) (numerical results can be found in Supplementary Table S1). At equimolarity (1:1) the median DFRET value of EGFP-PTS1(SCP2) was slightly but significantly higher than that of EGFP-SCP2, whereas the median DFRET of EGFP-SCPx was markedly lower, reflecting the larger size of SCPx and thus a lower DFRETmax. This interpretation is supported at higher acc:don ratios and by plotting all cells to depict saturation curves and to visualize their slopes and plateau level (Figure 2D). Comparing results of three different experiments revealed consistent overall patterns (Figure 2E). However, neither of these depictions of primary data is able to disentangle the contributions of different affinities (KDs) and different protein sizes (DFRETmax), which are reflected by different slopes and plateau levels of the saturation curves, respectively. However, when applying the fitting algorithm (Hochreiter et al., 2020) to infer measures of interaction strength (apparent KD, KD^app^)^1^ and DFRETmax (Figure 2F), we found that the predicted affinity of SCP2 was significantly higher than of the isolated PTS1 alone [numerical results can be found in Supplementary Table S2(overview)], whereas the affinity of SCPx appeared drastically lower. Moreover, the size of the donor molecules was reflected by the predicted DFRETmax values, as the PTS1 peptide has the highest and the large SCPx the lowest DFRETmax.

Next, we took advantage of this method to investigate the effect of two point mutations in the PTS1 of full-length SCP2 on its affinity to PEX5. On the one hand, the characteristic leucine at the ultimate position was substituted by the biophysically similar valine [AKL→AKV], which is hardly found in naturally occurring PTS1 and on the other hand the small residue glycine at position 139 directly involved in PEX5-binding (Stanley et al., 2006) was substituted by leucine (G139L, GNAKL). When flowFRET experiments were performed using SCP2, SCP2(AKL→AKV) and SCP2(G139L) we found that the median DFRET values of populations with equimolar acc:don ratio were markedly lower for AKV, but only slightly reduced for G139L (Figure 2G) and only the curve progression of SCP2(AKL→AKV) showed a clearly reduced slope in the ascending phase (Figure 2H). Comparing three different experiments showed a similar pattern (Figure 2I) and extracting KD1^app^s by fitting showed a clear increase [AKL→AKV] reflecting a lower interaction strength, whereas [G139L] did not significantly change the KD^app^ (Figure 2J).

Moreover, we explored the reliability of flowFRET for low-affinity binding partners. For that purpose, we studied the interaction between PEX5 (TPR) and the low-affinity PTS1 of human alanine-glyoxylate aminotransferase (AGXT) (PTS1 (AGXT)) (Ghosh and Berg, 2010) using the interaction with the full-length AGXT protein as comparison. Due to the low affinity of PTS1 (AGXT) the median DFRET of subpopulations was lower than for PTS1(SCP2) (*cf*.Figure 1A) (Figure 2K) and the saturation curve steadily increased even at high acc:don ratios without reaching the plateau level (Figure 2L). The higher binding strength of full-length AGXT compared to PTS1 (AGXT) is reflected by a higher median DFRET value of subpopulations with equimolar acc:don ratio in spite of a lower expected saturation level (DFRETmax) (Figure 2K) and a steeper ascend of the saturation curve (Figure 2L). Multiple independent experiments showed similar patterns with consistently low median DFRET values (Figure 2M). Although the median DFRET values of subpopulations and the curve progression clearly indicate PEX5 binding for PTS1 (AGXT), extracting numerical values for KD^app^ and DFRETmax by the fitting algorithm shows highly variable results due to the low affinity of the peptide, which exceeds the detrimental effects of the comparably larger size of the full-length protein (Figure 2N). Altogether, these results confirmed the applicability of the method for small proteins, but also demonstrated its limitations for the study of larger proteins and binding partners with low affinity.

### 3.2 Life cell competition experiments to circumvent problems due to low affinity or large protein size

To circumvent these limitations, we took advantage of the observation that ectopically expressed PTS1-carrying proteins exert a competitive effect on the interaction between mCherry-PEX5 (TPR) and EGFP-PTS1, which is reflected by a reduction of the DFRET values obtained by flowFRET (Hochreiter et al., 2020). When acting as a competitor, neither a large protein size nor a low affinity to the acceptor should be detrimental for FRET-measurements as these competitor proteins do not directly participate in measuring FRET efficiency but only modify the DFRET value of a cell. Tagging competitor proteins with the fluorescent protein Cerulean allows the estimation of the cellular level of competitor protein, which is essential to establish a quantifying fitting algorithm for extracting critical parameters of the competition. For a computational analysis of data sets obtained by flowFRET competition experiments, we had to solve the mathematical problem of this three-component system and to generate a computational program to infer the key parameters of the competition, namely KD1^app^ (donor-acceptor), DFRETmax, and KD2^app^ (competitor-acceptor). For that purpose, we adapted the analytic solution of the theoretical competition problem (Wang, 1995) and implemented it into a novel fitting program (*cf*. materials and methods). For internal normalization of the values in the Cerulean channel and to estimate the amount of competitor in a cell, we took advantage of the fusion protein mCherry-Cerulean and calculated proper conversion factor estimates (Hochreiter et al., 2020).

**FIGURE 3:**
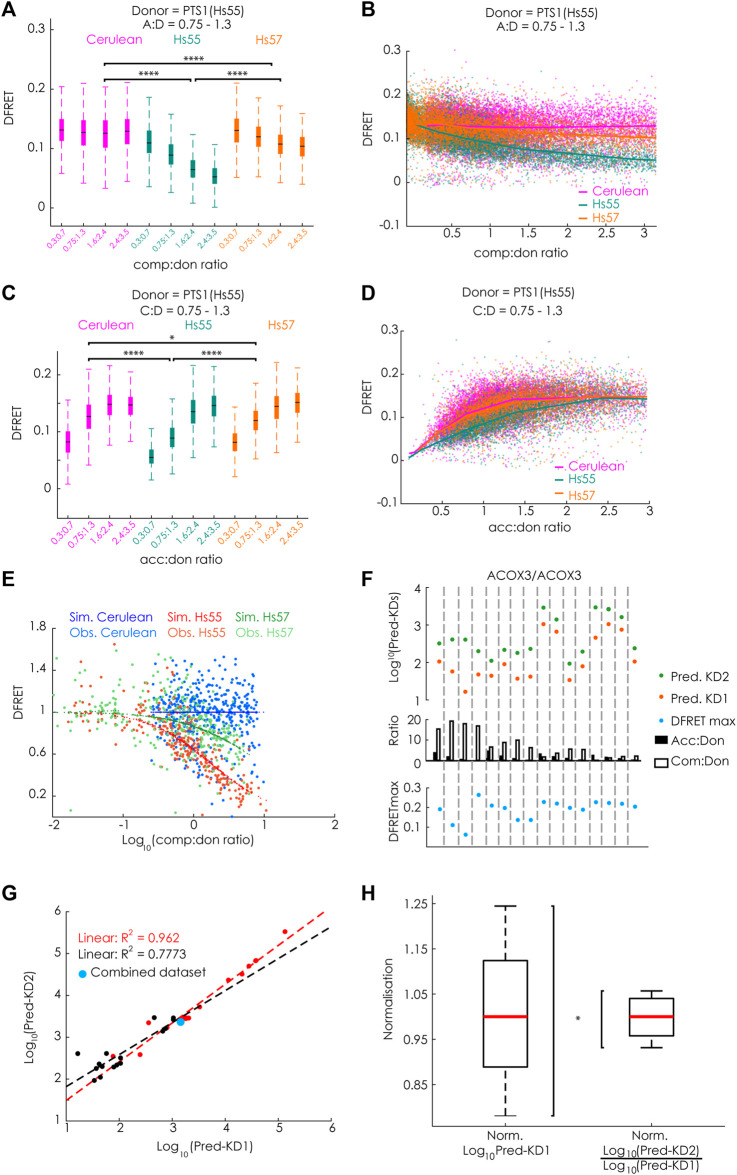
FlowFRET allows a competition assay in living cells: pex5^−/−^ MEF expressing mCherry-PEX5(TPR) as acceptor and various donor and competitor molecules were analyzed by flow cytometry to measure a large number of cells; **(A–E)** donor: EGFP-PTS1(Hs55), competitor: Cerulean, Cerulean-PTS1(Hs55) or Cerulean-PTS1(Hs57); **(A)** comparison of DFRET values of subpopulations of cells sharing an acc:don ratio of 0,75 < x < 1,3 and different comp:don ratios, **(B)** DFRET decay curves (increasing comp:don) of all cells sharing an acc:don ratio of 0,75 < x < 1,3; **(C)** comparison of DFRET values of subpopulations of cells sharing an comp:don ratio of 0,75 < x < 1,3 and different acc:don ratios; **(D)** plotting DFRET values of all cells sharing a comp:don ratio between 0,75 < x < 1,3 to generate saturation curves (increasing acc:don); **(E)** the results the fitting algorithm for Hs55 (KD1^app^: 14076, KD2^app^: 26258 and DFRETmax: 0.153) and Hs57 (KD1^app^: 14400, KD2^app^: 199636 and DFRETmax: 0.153) were used to calculate for each cell based on the values for the donor, acceptor and competitor level the expected values for DFRET in the presence or absence of competitor, the ration of the values as % were plotted against the Log_10_ of the comp:don ratio to obtain a traditional inhibition curve (blue, green and red) and the corresponding ration of the observed values are depicted as with the same colors; **(F–H)** donor: EGFP-PTS1(ACOX3), competitor: Cerulean-PTS1 (ACOX3): **(F)** cell populations obtained by independent transformations with different relative amouts of expression plasmids for donor and acceptor display different medians of acc:don (black) or comp:don (white) ratios (middle), when extracting by the fitting algorithm KD1^app^ (Pred.KD1^app^, orange), KD2^app^ (Pred.KD2^app^, green) and DFRETmax (blue) values for each of these different data we obtained results of marked variability; **(G)** however, when plotting the Log_10_(Pred-KD1^app^) against the Log_10_(Pred-KD2^app^) the values were highly correlated (black line), which became even stronger (orange line) when the fitting was performed with a predefined DFRETmax (0.226), which was obtained by predicting these values from a united data set composed of all 16 data sets (blue point); **(H)** compiling the results of fittings applied to all available data sets obtained either by bimolecular flowFRET (donor: PTS1(ACOX3)) or competitive flowFRET (donor and acceptor PTS1(ACOX3)) the normalized variability (s/median) was markedly smaller for the latter expressed as Log_10_(KD2^app^)/Log_10_ (KD1^app^) compared to the first expressed as Log_10_ (KD1^app^). Statistics: A&C: box blots with quartils and ranges, multicomparison “Tukey-Kramer” for cells sharing comp:don ratios between 1,6 < x < 2,4 (A, Cerulean vs. Hs55 < 1e-100, Cerulean vs. Hs57 < 1e-100, Hs55 vs. Hs57 < 1e-100) or acc:don rations between 0,75 < x< 1,3 (C, Cerulean vs. Hs55 < 1e-100; Cerulean vs. Hs57 = 0.0305; Hs55 vs. Hs57 < 4.825e-07); **(H)** Two-sample F-test for equal variances (*p* = 0.0356).

To verify the mathematical approach, we took advantage of available large data sets originating from experiments, in which the apparent interaction strength between mCherry-PEX5 (TPR) and EGFP-PTS1, harboring the arbitrary PTS1-peptide Hs55 (EGFP-PTS1(Hs55) (Lametschwandtner et al., 1998)), was modulated by Cerulean-tagged PTS1 peptides, encoding either the same high affinity PTS1 Hs55 or the low affinity PTS1 Hs57, using Cerulean alone as a negative control, which should not affect the interaction strength [(Hochreiter et al., 2020), Figure 5]. To estimate the effect size of competition, we first preselected cells sharing an acc:don ration around one (0.75 < x < 1.3) and compared the changes in the median DFRET values of subpopulations each sharing narrow, but well-defined competitor to donor (comp:don) ranges. By this means, we were able to visualize differences between samples expressing a competitor or not, but also between competitors with high (Hs55) or low (Hs57) affinity (Figure 3A). The overall shape of the curve progression of the decay curve (increasing comp:don) also differed clearly between high and low affinity competitors (Figure 3B). Similarly, comparing subpopulations sharing a comp:don ratio of one, but differing in the acc:don ratio showed clear differences as well (Figure 3C) and also the saturation curve (increasing acc:don) showed clear differences (Figure 3D). When analyzing these data sets with our newly generated algorithm we obtained values for KD1^app^, KD2^app^ and DFRETmax, which were comparable to those obtained by bimolecular flowFRET. Next, we used these values to calculate the fraction of donor molecules engaged in acceptor-donor complexes (acc.don) in the presence or absence of competitor and estimated the expected reduction of DFRET due to competition for each data point. Plotting these values against the comp:don ratio depicted as Log_10_ showed a decay curve very similar to characteristic curves of biochemical competition experiments, which supported the validity of the mathematical solution (Figure 3E).

### 3.3 Computational exploration of the fitting algorithm

To further validate the power of this life-cell competition approach, we systematically investigated different aspects of this method to explore possible limitations. Although Cerulean-tagged proteins do not affect FRET efficiency measurements between EGFP and mCherry, the energy transfer from Cerulean-tagged cargo proteins to mCherry-PEX5(TPR) reduces the measured intensity in the Cerulean channel. To estimate the consequences of a comparably small underestimation of the Cerulean level, we calculated the effective changes for the results of competition experiments using EGFP-PTS1(Hs55) as donor and Cerulean-PTS1(Hs55) as competitor (Hs55/Hs55), because a competitor with a short, but high-affinity peptide should present with the largest deviations (for details see Supplementary File S1A). We found that the mean error size for Cerulean intensity values was around 5.87 ± 2.89% and when using corrected values for the Cerulean channel the changes in acc.don complexes, which directly relates to the error of the DFRET value, were about 1.27 ± 0.8%. Subjecting the corrected data set to the fitting protocol resulted in very similar values for KD1^app^ (4% lower), KD2^app^ (1.7% lower) values and the DFRETmax level (0.65% lower), which is negligible compared to the variation obtained between the results of different experiments. Moreover, we validated that the output of the fitting was robust against a slight misjudgment of the background value of the FRET-channel. When all data points of a given data set, either Hs55/Hs55 or Hs55/Hs57, were incrementally in- or decreased and these modified data sets were refitted (Supplementary File S1B), we found that the values of KD1^app^ and KD2^app^ expressed as Log_10_ often remained unchanged and only occasionally KD1^app^ and KD2^app^ changed, but then in a similar manner. In contrast, the extracted values for DFRETmax followed the incremental changes in the DFRET values demonstrating the robustness of the fitting algorithm to slight inconsistencies in the definition of the DFRET-background level, next to the expected changes in DFRETmax. Furthermore, we investigated in how far the reliability of the fitting procedure was affected by the sample size. For that purpose, we randomly drew subpopulations of different size from the data set Hs55/Hs55, and independently fitted ten subpopulations of the same size to compare the inferred values for KD1^app^ and KD2^app^ and to estimate their variance. We found that already at sample sizes of 250 data points the median of ten samples was close to the result obtained by fitting the entire data set, but a low variance among different subsets was observed only when at least 1,000 data points per subset were used (for details see Supplementary File S1C). Moreover, we investigate the interdependence of the three fitted parameters, KD1^app^, KD2^app^ and DFRETmax by analyzing the changes in the mean error size (mean square error, MSE) upon incrementally changing and predefining one of these values and resubmitting the data set to another fitting (Supplementary File S1D **)**. When the two other parameters were predefined at the original values the MSE drastically increased in all cases, whereas only minor changes were observed when the values for the other parameters were free to readapt. Especially KD1^app^ and KD2^app^ very effectively compensated aberration of the other value, which corroborates the capability of the fitting procedure to recapitulate the tight relation between the slope of the decay curve and the KD2/KD1 ratio. This suggested that the ratio of KD2^app^ and KD1^app^ is a more robust measure of the relative binding strength obtained by this fitting procedure. Next, we tested the effect of marked changes in the composition of a data set with regard to the relative amounts of acceptor, donor and competitor expressed in the cells. For that purpose, we repeated the fitting of the competition experiment using the data sets Hs55/Hs55 and Hs55/Hs57, but considered only cells within predefined ranges of acc:don and comp:don (Supplementary File S1E). We found that the results of fittings using subsamples that cover exclusively very low or very high acc:don ratios and subsamples enclosing only high comp:don ratios inferred markedly different values for KD1^app^ and KD2^app^ even when expressed as Log_10_(KD^app^). However, importantly, the two values consistently changed into the same direction supporting the hypothesis that the Log_10_(KD2^app^)/Log_10_(KD1^app^) is more reliable. As these subdivisions were artificially generated by strict threshold, we independently confirmed this observation by using donor and competitor proteins equipped with the PTS1 of ACOX3 (PTS1(ACOX3)), ACOX3/ACOX3. Cells were transfected with expression plasmids encoding donor, acceptor and competitor in widely differing combinations and each of these transformations was used as an independent sample (Figures 3F,G). We found that overall high acc:don ratios were associated with high median DFRET values and high comp:don levels with low median DFRET values (Supplementary Figure S2A). Moreover, uniting all independently transformed cells (ACOX3/ACOX3_1–16) to one large data set, subdividing this data set according to the comp:don ratio and independently subjecting these subpopulations to the bimolecular fitting verified a direct correlation between the comp:don ratio and the inferred value for KD1^app^ (Supplementary Figure S2B), which confirmed the internal coherence of the different subsets. Moreover, when fitting the original data sets independently the predicted values for Log_10_(KD1^app^) and Log_10_(KD2^app^) varied markedly between the samples (Figure 3F), but the difference between these values and thus the ratio of KD2^app^ to KD1^app^ was more similar. When plotting Log_10_(KD2^app^) against Log_10_(KD1^app^) we found a high correlation (R^2^ = 0.773) (Figure 3G), which was further increased (R^2^ = 0.962) when for the fitting identical DFRETmax values were predefined for all samples and thus compensatory effects related to the adjustment of the DFRETmax value were excluded. These results confirmed that a certain bias in the inference of KD1^app^ and KD2^app^ caused by a biased composition of the data set is compensated by the concomitant deviation of both values and importantly, the values for KD1^app^ and KD2^app^ inferred from the united large data set is located at the center of the two curves (blue point). Thus, the internal normalization renders this fitting approach more robust. To verify this hypothesis, we analyzed all available results of independent flowFRET experiments studying the interaction between PEX5(TPR) and the PTS1(ACOX3) either by traditional bimolecular measurements between mCherry-PEX5(TPR) and EGFP-PTS1(ACOX3) or competition experiments in which Cerulean-PTS1(ACOX3) was included (Figure 3H). We found that the normalized variance was significantly lower for the competition experiments, which confirmed that the Log_10_(KD2^app^)/Log_10_(KD1^app^) ratio is a more robust and reliable measure for the apparent interaction strength.

### 3.4 Verifying the power of the live-cell flowFRET competition assay

**FIGURE 4:**
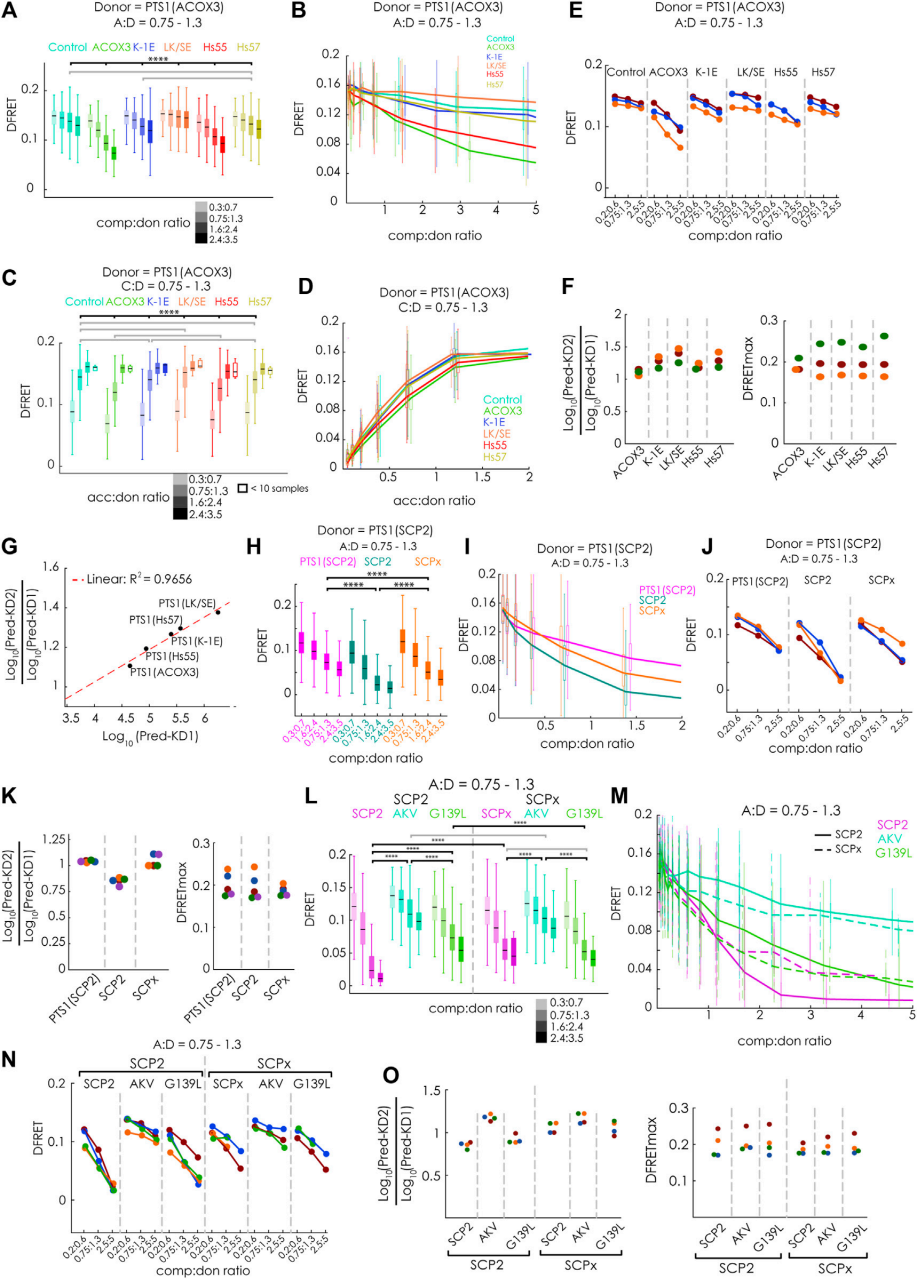
FlowFRET competition assays in living cells allow the discrimination of affinities and is applicable to large proteins: pex5^−/−^ MEF expressing mCherry-PEX5(TPR) as acceptor and various donor and competitor molecules were analyzed by flow cytometry to obtain a large number of cells: **(A–G)** donor: EGFP-PTS1 (ACOX3), competitor: various Cerulean-PTS1 proteins harboring the PTS1 motifs of Hs55, Hs57, ACOX3, ACOX3_K-1E, ACOX3_LK/SE or Cerulean alone; **(H–K)** donor EGFP-PTS1(SCP2), competitor Cerulean-PTS1(SCP2), Cerulean-SCP2 or Cerulean-SCPx; **(L–O)** donor EGFP-PTS1(SCP2), competitor Cerulean-SCP2, Cerulean-SCP2(AKL→AKV) or Cerulean-SCP2(G139L), Cerulean-SCPx, Cerulean-SCPx (AKL→AKV) or Cerulean-SCPx(G139L); to compare cells expressing different competitor proteins either **(A,H,L)** the median DFRET values of subpopulations sharing an acc:don ratio of 0,75 < x < 1,3 and different competitor to donor ratios or **(B,I,M)** the DFRET decay curves (increasing comp:don) of cells sharing an acc:don ratio of 0,75 < x < 1,3 are depicted; alternatively, **(C)** the median DFRET values of subpopulations sharing a comp:don ratio of 0,75 < x < 1,3 and different acc:don ratios or **(D)** the DFRET saturation curves (increasing acc:don) in the presence of a comp:donor ratio of 0,75 < x < 1,3 are depicted; **(E,J,N)** the pattern of a decay of median DFRET values with increasing median comp:don ratios is comparable between three different experiments and **(F,K,O)** the results of the fitting algorithm (Pred-KD1^app^, Pred-KD2^app^ and DFRETmax) are plotted as ratio Log_10_(Pred.-KD2)/Log_10_(Pred.-KD1) (left side) and as DFRETmax (right side); **(G)** plotting the affinities of these peptides as Log_10_(KD) from our previous study (Hochreiter et al., 2020) against the Log_10_(Pred.-KD2)/Log_10_(Pred.-KD1) from the competition method we obtained a high correlation. Statistics: A,C,H,L: box blots with quartiles and ranges, multicomparison “Tukey-Kramer” for cells sharing acc:don rations between 0,75 < x < 1,3 and comp:don ratios between 1,6 < x < 2,4 (A,H,L) or cells sharing comp:don rations between 0,75 < x < 1,3 and acc:don ratios between 0,75 < x < 1,3 **(C)**, predictions **(F)** were analyzed by a bootstrapping method (details of the statistics *cf.* Supplementary Table S2).

Next, we evaluated, whether our new method can discriminate competitors with different binding strengths and measure the interaction strength of large binding proteins. For that purpose, we first systematically investigated the modulatory effect of different competitor proteins on the interaction between mCherry-PEX5(TPR) and EGFP-PTS1(ACOX3). We used Cerulean extended by the PTS1 of ACOX3 or two variants thereof with lower affinity (K-1E or LK/SE) or the PTS1 of Hs55 or Hs57 (Hochreiter et al., 2020). We analyzed these data sets by first selecting cells with an acc:comp ratio of about one and depicting the effect of increasing comp:don ratio on the median DFRET value either by comparing subpopulations with different comp:don ratio (Figure 4A) or by depicting decay curves (Figure 4B). Alternatively, we selected cells with a comp:don ratio around one and followed the ascending DFRET values with increasing acc:don ratios at the level of subpopulations (Figure 4C) or as saturation curves (Figure 4D). Depicting subpopulations revealed differences in the median DFRET with a comp:don ratio around two or an acc:don ratio of about one (Figure 4A&3C) and the decay and saturation curves displayed different slopes (Figures 4B,D). The relative fall of the decay curves differed among the competitors (Figure 4C) and their ranking reflected the affinity of the competitor peptides, with ACOX3 and Hs55 being high affinity, Hs57 and K-1E being intermediate affinity and LK/SE being a low-affinity binders [based on previous experiments (Hochreiter et al., 2020)]. Moreover, the pattern of DFRET decay of the selected populations was similar among different independent experiments (Figure 4E). Conversely, the apparent slope in the ascending phase of the saturation curve in the presence of a competitor decreased with the competitor´s increasing affinity (Figure 4D). When these data sets were subjected to the fitting algorithm and the extracted values were plotted as ratio Log_10_(KD2^app^)/Log_10_(KD1^app^), we obtained consistent results (Figure 4F, left side) and the ranking of these values reflected the relative slope of the decay curves. In addition, by plotting the average of the Log_10_(KD2^app^)/Log_10_(KD1^app^) values for the peptides against the values obtained by bimolecular measurements [(Hochreiter et al., 2020); inverse values as K_a_^app^ had been used) we obtained a high correlation (Figure 4G). However, when inspecting the predicted DFRETmax of these data sets (Figure 4F, right side], these values were relatively similar for each experiment reflecting the use of the identical donor but differed more between the experiments. These results demonstrate that the competition assay can effectively discriminate the affinity of different PTS1 peptides and that for peptides the results are in good agreement with the results of bimolecular measurements.

To verify that the competition approach is also capable of improving the performance for large proteins less accessible by bimolecular measurements due to limitations by the low DFRETmax value (cf.Figure 1B), we performed flowFRET competition experiments using EGFP-PTS1(SCP2) as donor and Cerulean-PTS1(SPC2), Cerulean-SCP2 or Cerulean-SCPx as competitors (Figures 4H–K). Comparing subsets with acc:don ratios around one and different comp:don ratios, the median DFRET value of cells expressing SCP2 was lower than those with PTS1(SCP2), reflecting a higher affinity of SCP2, whereas SCPx caused an intermediate reduction (Figure 4H). However, in contrast to Figure 1, the median DFRET values at low comp:don levels were very similar between all samples and thus are not affected by the size of the competitor. The negative slopes of the decay curves were also steeper for Cerulean-SCP2 than for those caused by Cerulean-SCPx or Cerulean-PTS1(SCP2) (Figure 4I), and comparison of three different experiments showed similar overall shapes, although the decay curve due to SCPx appears more similar to that caused by PTS1(SCPx) (Figure 4J). Upon fitting these data sets, we found that PTS1(SCP2) presents with a Log_10_(KD2^app^)/Log_10_(KD1^app^) value around one, which reflects the identity of the PTS1 in donor and competitor protein, whereas SCP2 shows a lower Log_10_(KD2^app^)/Log_10_(KD1^app^) ratio and thus a higher affinity than the PTS1 of the donor (Figure 4K, left part). The Log_10_(KD2^app^)/Log_10_(KD1^app^) ratio for SCPx was also around one and resembles the isolated PTS1. Again, the predicted values for DFRETmax were similar between different samples within the same experiment reflecting the identity of the donor molecules but displayed higher variability between the experiments (Figure 4K, right part).

Finally, we addressed the question, whether differences in the affinities can be reliably measured for such large proteins as well. For that purpose, we repeated the competition experiments but compared the effects of the mutations [AKL→AKV] and [G139L] (*cf*. Figure 1) on the affinity of SCP2 and SCPx (Figures 4L–O). We found, at the level of individual subpopulations, that the effectivity of competition was markedly reduced by the mutation [AKL→AKV], whereas that of [G139L] was clearly reduced for SCP2 but less so for SCPx (Figure 4L), which was also reflected in the early phase of the decay curve (Figure 4M). Comparing three repetitions of these experiments show very similar patterns (Figure 4N), and the results of extracting key parameter from these data sets by the fitting algorithm were consistent (Figure 4O). On the one hand, the Log_10_(KD2^app^)/Log_10_(KD1^app^) ratios were larger for the mutation [–AKV], whereas no apparent differences were found for the mutation [G139L], but on the other hand, the results appear highly consistent between the different experiments, but also regarding the predicted DFRETmax for each experiment. These results verify that this competition-based method is well-suited to study complexes including large proteins.

### 3.5 Investigation of full-length cargo-binding by PEX5 (TPR)

**FIGURE 5:**
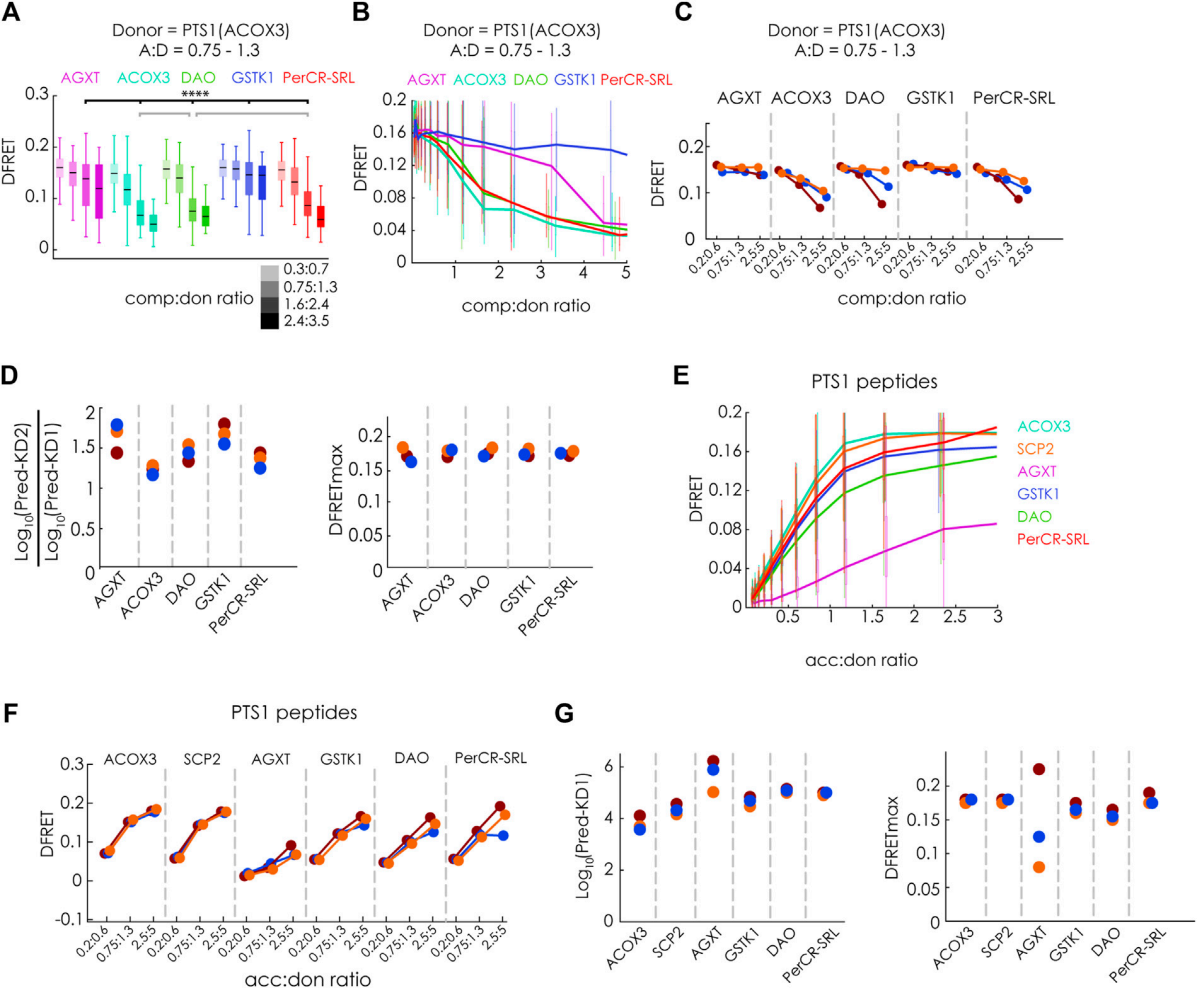
Studying the binding of full-length PTS1-carrying proteins to PEX5 by flowFRET competition experiments: pex5^−/−^ MEF expressing mCherry-PEX5(TPR) as acceptor and various donor and competitor molecules were analyzed by flow cytometry to obtain a large number of cells **(A–D)** donor: EGFP-PTS1(ACOX3), competitor: Cerulean-tagged full length proteins of hAGXT, hACOX3, hDAO, hGSTK1, pig PerCR; **(A)** the median DFRET values of subpopulations sharing an acc:don ratio of 0,75 < x < 1,3 and different competitor to donor ratios or **(B)** the DFRET decay curves (increasing comp:don) of cells sharing an acc:don ratio of 0,75 < x < 1,3 are depicted (*cf*. Figures 3A,B); **(C)** the pattern of the decay curves is comparable between three different experiments and **(D)** the results of the fitting (Pred.-KD1, Pred.-KD2 and DFRETmax) are plotted as ratio of Log_10_ (Pred.-KD2)/Log_10_ (Pred.-KD1) or as DFRETmax; **(E–G)** donor: EGFP-PTS1 variants harboring the PTS1 of hAGXT, hACOX3, hDAO, hGSTK1, pig PerCR; **(E)** DFRET saturation curves (increasing acc:don); **(F)** when comparing subpopulations sharing the same acc:don ratio the pattern is comparable between three different experiments and **(G)** results of the fitting algorithm (Pred.-KD1, and DFRETmax) are plotted as Log_10_(Pred.-KD1) (left side) and DFRETmax (right side). Statistics: **(A)** box blots with quartiles and ranges, multicomparison “Tukey-Kramer” for cells sharing acc:don rations between 0,75 < x < 1,3 and comp:don ratios between 1,6 < x < 2,4, predictions **(D)** and **(G)** were analyzed by a bootstrapping method (details of the statistics *cf.* Supplementary Table S2).

After these confirmations, we investigated the interaction of PEX5(TPR) with several PTS1-carrying full-length proteins, namely human AGXT (Fodor et al., 2012), acyl-CoA oxidase 3 (ACOX3) (Van Veldhoven et al., 1994), D-amino acid oxidase (DAO) (Sacchi et al., 2011) and Glutathione-S-transferase kappa-1 (GSTK1) (Wang et al., 2013) as well as pig peroxisomal carbonyl-reductase (PerCR-SRL) (Tanaka et al., 2008). We used mCherry-PEX5(TPR) and EGFP-PTS1 (ACOX3) as FRET-pair and co-expressed the above-mentioned proteins as fusion proteins with Cerulean. We found that in individual experiments, the median DFRET values of subpopulations with very similar acc:don and comp:don ratios were consistently reduced in a dose-dependent manner (Figure 5A). This effect appeared smaller than for SCP2 (*cf*. Figure 3H), but in the latter case EGFP-PTS1(SCP2) with a lower affinity was used as donor. The curve progressions showed a clear decay for ACOX3, DAO and PerCR-SRL, whereas the slope was less pronounced for AGXT and minimal for GSTK1 (Figure 5B). These results were consistently found in three independent experiments (Figure 5C). When the data sets were subjected to the fitting algorithm, Log_10_(KD2^app^)/Log_10_(KD1^app^) ratios were rather similar between the experiments as were the values for the predicted DFRETmax values (Figure 5D). Although clear differences in the competitive effectivity for the different full-length proteins were observed, the values for Log_10_(KD2^app^)/Log_10_(KD1^app^) were markedly larger than one, suggesting a drastically lower affinity than the donor. However, again, a direct comparison with results obtained for SCP2 (*cf.* Figures 3G–N) is not possible, because in these experiments EGFP-PTS1(ACOX3) served as donor, whereas in the latter experiments PTS1(SCP2). Thus, we compared these results with competition experiments using EGFP-PTS1(SCP2) as donor and found that the effectivity of competition was higher (Supplementary Figure S3A) and the Log_10_(KD2^app^)/Log_10_(KD1^app^) ratio for the same full-length proteins was lower (Supplementary Figure S3B). Importantly, the relative binding strength was retained between these experiments as the results of both experiments showed good correlation (Supplementary Figure S3C), but nonetheless, the Log_10_(KD2^app^)/Log_10_(KD1^app^) ratios were markedly larger than one suggesting that these full-length proteins did not bind as strong to PEX5 as did SCP2.

To better relate these results to the affinity of isolated PTS1 peptides within living cells, we independently investigated the affinity of different EGFP-PTS1 variants harboring the last 12 amino acids of the studied proteins and mCherry-PEX5(TPR) by bimolecular flowFRET experiments. We found saturation curves with clearly different slopes (Figure 5E). In three independent experiments we consistently found high DFRET signals in subfractions sharing the same acc:don ratio except for the PTS1 of AGXT (Figure 5F). When extracting KD1^app^ and DFRETmax by the fitting algorithm (Figure 5G), we obtained different affinities ranging across more than an order of magnitude as described previously (Ghosh and Berg, 2010; Hochreiter et al., 2020), whereas the inferred DFRETmax values were similar except for the high variability of DFRETmax of PTS1 (AGXT), similar to our previous results (*cf*.Figure 1L).

Overall, the ranking of the affinities of these 12aa long peptides fused to EGFP was similar to that obtained by biophysical measurements of isolated PEX5 and 6aa peptides (Ghosh and Berg, 2010) and correlated with full-length proteins except for the peptide of GSTK1 (12aa), which appeared with intermediate affinity binding peptide in our assay but displayed comparably weak binding as 6aa peptide and very weak binding as full-length protein (*cf*. Figures 4C,D). However, although the relative affinities of full-length proteins and isolated PTS1 peptides was similar except for GSTK1 and consistent between experiments using different donors, the comparably low affinity of full-length proteins was surprising, because the PTS1 peptides of SCP2 and AGXT had a clearly lower affinity to PEX5 than the corresponding full-length proteins (*Cf*.Figure 1). In the latter cases the stronger binding can probably be retraced to additional interaction domains with PEX5 (Stanley et al., 2006; Fodor et al., 2012) although overall AGXT bound with markedly lower affinity than SCP2. We assumed that the oligomerization status of the investigated proteins might affect the binding strength, because in the case of human catalase tetramerization and PEX5 binding were found to compete (Freitas et al., 2011), whereas the previously studied SCP2 occurs exclusively as a monomeric protein and AGXT dimers can effectively bind to PEX5 (Fodor et al., 2012). Among the studied proteins DAO and GSTK1 have been described as dimers (Kawazoe et al., 2006; Wang et al., 2011), PerCR as tetramer (Tanaka et al., 2008) and ACOX3 even as octamer (Van Veldhoven et al., 1994). Provided that some fully folded proteins expose their C-terminus less effectively upon oligomerization, mCherry-PEX5(TPR) used in our study might not be able to effectively compete for the PTS1, because only a PEX5-variant in which the TPR-domain is extended at its N-terminus was able to disassemble catalase oligomers (Freitas et al., 2011).

### 3.6 Investigating PEX5(N-TPR)

**FIGURE 6:**
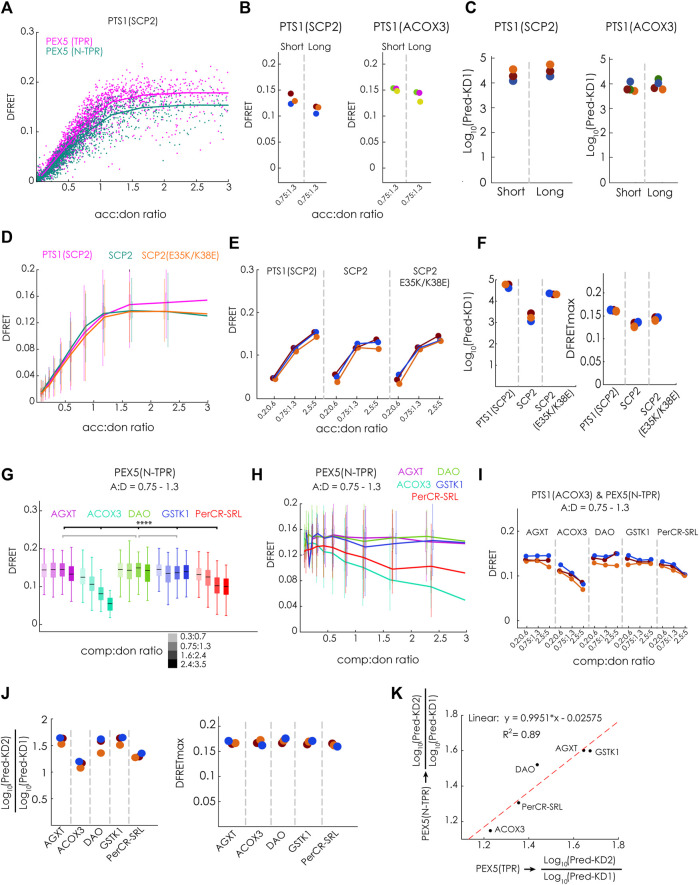
N-terminal extension of the PEX5-TPR domain (PEX5(N-TPR)) has a similar affinity to full-length PT1-carrying cargo proteins: **(A–F)** Bimolecular flowFRET experiments in pex5^−/−^ MEF: **(A)** saturation curves of cells expression EGFP-PTS1(SCP2) as donor and either mCherry-PEX5(TPR) or mCherry-PEX5(N-TPR) as acceptor show different plateau levels; **(B)** the median DFRET value of populations sharing an acc:don ratio (0,75 < x < 1,3) is higher for mCherry-PEX5(TPR) than for mCherry-PEX5(N-TPR) when comparing repeated experiments and using either EGFP-PTS1(SCP2) or EGFP-PTS1(ACOX3) as binding partners, **(C)** the results of fitting these data sets (Pred-KD1^app^) are plotted; **(D)** saturation curves for mCherry-PEX5(N-TPR) as donor and either EGFP-PTS1(SCP2), EGFP-SCP2 or EGFP-SCP2(E35K/K38E); **(E)** overall pattern of median DFRET values for different acc:don subpopulations is consistently observed in three different experiments of the one described in **(D)**; **(F)** the results of the fitting algorithm (Pred-KD1^app^ and DFRETmax) are plotted for the interactions in **(D)** and **(E)**; **(G–K)** flowFRET competition experiments: pex5^−/−^ MEF expressing mCherry-PEX5(N-TPR) as acceptor, EGFP-PTS1(ACOX3) as donor and different Cerulean-tagged full-length proteins as competitor molecules were analyzed by flow cytometry to obtain a large number of cells, **(G)** median DFRET values of subpopulations sharing an acc:don ratio of 0,75 < x < 1,3 and different competitor to donor ratios or **(H)** DFRET decay curves (increasing comp:don ratios) of cells sharing an acc:don ratio of 0,75 < x < 1,3 are depicted; **(I)** the pattern of progressive reduction in the median DFRET value of populations with increasing comp:don ratios is consistently observed in three different experiments; **(J)** the results of the fitting algorithm (Pred-KD1, Pred-KD2 and DFRETmax) are plotted as ratio Log_10_(Pred.-KD2)/Log_10_(Pred.-KD1) or as DFRETmax, **(K)** plotting the Log_10_(Pred.-KD2)/Log_10_(Pred.KD1) values for the interaction of the same competitor molecules with either PEX5(TPR) (x-axis) or PEX5(N-TPR) (y-axis) are highly correlated. Statistics: **(G)** box blots with quartiles and ranges, multicomparison “Tukey-Kramer” for cells sharing acc:don rations between 0,75 < x < 1,3 and comp:don ratios between 1,6 < x < 2,4, predictions **(J)** were analyzed by a bootstrapping method (details of the statistics *cf.* Supplementary Table S2).

To test the hypothesis that a PEX5-variant with an N-terminal elongation (N-TPR) has markedly higher affinities to these full-length PTS1-carrying proteins, because it is able to disassemble oligomeric protein complexes preventing PEX5 (TPR) from binding, this protein, PEX5(N-TPR), was studied in detail. We first performed a flowFRET experiment to study the interaction between EGFP-PTS1(SCP2) and either PEX5(TPR) or PEX5(N-TPR) and found that the slope of the saturation curves appeared very similar. However, in the presence of the N-terminal extension the DFRETmax level was reduced, which was expected and reflected the larger distance between EGFP and mCherry in the complex (Figure 6A). This pattern was also found when comparing median DFRET values at equimolar acc:don ratio of independent experiments and different PTS1(SCP2 and ACOX) as binding partners, although the difference was less clear for PTS1(ACOX3) (Figure 6B). Applying the fitting algorithm to these data sets allowed the extraction of KD^app^, which appeared slightly higher for PEX5(N-TPR) for PTS1(SCP2), whereas no difference was found for PTS1 (ACOX3) (Figure 6C). Next, we investigated, whether PEX5(N-TPR) binds SCP2 with higher affinity than PTS1(SCP2) as well and studied the effect of inverting the charge of two positions in the N-terminal part of SCP2, namely glutamate 35 to lysine (E35K) and lysine 38 to glutamate (K38E), which are clearly separated from the PTS1 but in the 3D-structure were found to participate in an additional PEX5-binding domain (Stanley et al., 2006). When bimolecular flowFRET experiments were performed comparing EGFP-PTS1(SCP2), EGFP-SCP2 or EGFP-SCP2(E35K/K38E), we found that the slope of the saturation curve appears steeper for SCP2, whereas the plateau level was clearly higher for PTS1(SCP2) (Figure 6D). A similar pattern was observed in three independent experiments when plotting subpopulations with similar acc:don ratio (Figure 6E), but again the contribution of the affinity and the saturation level could not be disentangled (*cf*.Figure 1). However, when applying the fitting algorithm to the corresponding data sets independent values for KD1^app^ and DFRETmax were extracted (Figure 6F). The affinity of full-length SCP2 was estimated to be higher than an isolated PTS1 but introducing the point mutations E35K and K38E ablated this difference, whereas the DFRETmax values still reflected differences in protein size. These results suggests that the contribution of the additional interphase between SCP2 and PEX5 is abrogated in SCP2(E35K/K38E).

When we performed flowFRET competition experiments using mCherry-PEX5(N-TPR) together with the same PTS1-carrying full-length proteins as before (*cf.* Figure 4), we found that the median DFRET values of subpopulations sharing an acc:don ration around one and different comp:don ratios were markedly reduced in the presence of ACOX3 as competitor protein, intermediate effects were observed for PerCR-SRL, low effects for AGXT, and nearly no effect was found for DAO and GSTK1 (Figure 6G). This pattern was also supported by the decay curves (Figure 6H) and observed in three independent experiments (Figure 6I). Subjecting these data sets to the fitting algorithm allowed the extraction of very similar values for DFRETmax, whereas for the different competitor proteins different Log_10_(KD2^app^)/Log_10_(KD1^app^) ratios were obtained (Figure 6J). The pattern of Log_10_(KD2^app^)/Log_10_(KD1^app^) values for PEX5(N-TPR) resembled those for PEX5(TPR) (*cf*.Figure 4G), with a high correlation (R^2^ = 0,89) and a slope very close to one (Figure 6K).

Altogether, these results demonstrate that the affinity of full-length PTS1-carrying proteins to PEX5(TPR) and PEX5(N-TPR) is comparable and that the extension of the TPR domain at the N-terminus does not increase the apparent affinity for these proteins in living cells.

### 3.7 Modulatory role of the N-terminal domain of PEX14

**FIGURE 7:**
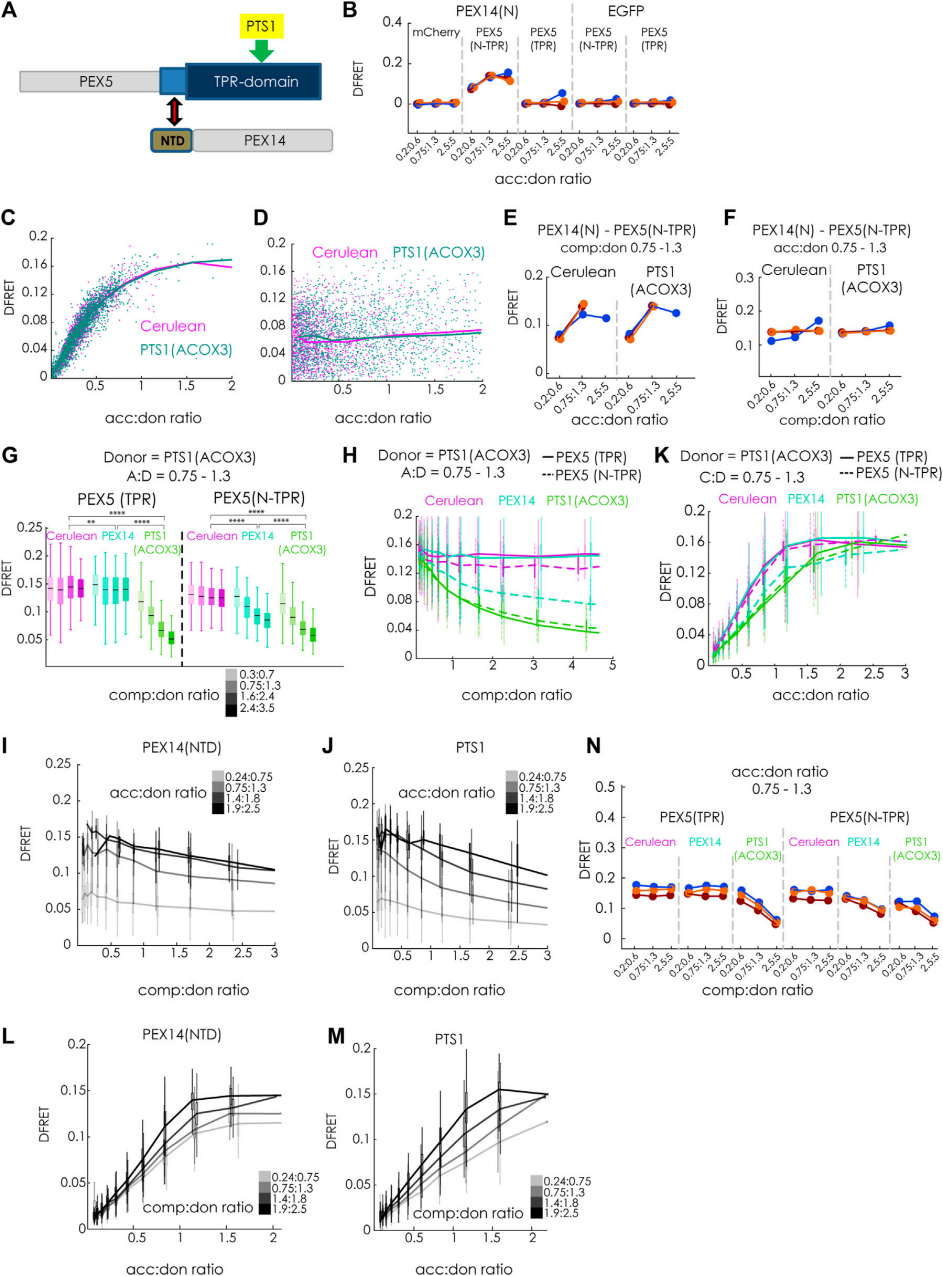
The N-terminal domain of PEX14 modulates the affinity of PEX5 and its cargo by a non-competitive mechanism: (A) Scheme: the interaction between the TPR-domain of PEX5 (blue) and the PTS1 (yellow) is modulated by the N-terminal domain (NTD, brown) of PEX14, binding to an N-terminal extension of the TPR-domain (bright blue); grey: parts of PEX5 and PEX14 not encoded in the test proteins; **(B)** bimolecular flowFRET experiments in pex5^−/−^ MEF verifying the interaction between PEX14(NTD)-EGFP and mCherry-PEX5(N-TPR), but not mCherry-PEX5(TPR) in living cells; **(C–F)** flowFRET experiments in pex5^−/−^ MEF: the interaction between PEX14(NTD)-EGFP and mCherry-PEX5(N-TPR) is not affected by the expression Cerulean-PTS1 compared to Cerulean alone, when depicted as saturation curve **(C)** or as dose-response curve with respect to Cerulean-PTS1 **(D)**; these results are consistently observed in three independent experiments; (**G–M**) flowFRET competition experiments: pex5^−/−^ MEF were transfected mCherry-PEX5(N-TPR) as acceptor and EGFP-PTS1 (ACOX3) as donor together with either PEX14(NTD)-Cerulean, Cerulean-PTS1 or Cerulean alone **(G)** the median DFRET values of subpopulations sharing an acc:don ratio of 0,75 < x < 1,3 and different competitor to donor ratios or **(H)** the DFRET decay curves (increasing comp:don) of all cells sharing an acc:don ratio of 0,75 < x < 1,3 are depicted; **(I)** and **(J)** comparison of decay curves due to PEX14(NTD)-Cerulean **(J)** or Cerulean-PTS1 **(K)** of cells sharing different acc:don ratios; **(K)** DFRET saturation curves (increasing acc:don) in the presence of a comp to donor ratio of 0,75 < x < 1,3 are depicted; **(L,M)** comparison of changes in the saturation curves due to PEX14(NTD)-Cerulean **(L)** or Cerulean-PTS1 **(M)** are depicted for different comp:don ratios; **(N)** the pattern of DFRET values of subpopulations sharing an acc:don ratio of 0,75 < x < 1,3 and different competitor to donor ratios is similar between three independent experiments. Statistics: **(G)** box blots with quartiles and ranges, multicomparison “Tukey-Kramer” for cells sharing acc:don rations between 0,75 < x < 1,3 and comp:don ratios between 1,6 < x < 2,4 (details of the statistics *cf.* Supplementary Table S2).

Finally, we investigated whether our method is capable of discriminating different modes of inhibiting protein-protein interaction, which are traditionally classified as competitive and non-competitive (or allosteric). For that purpose, we compared the inhibitory effects of the competitive inhibitor Cerulean-PTS1, with that of a putatively non-competitive modulator PEX14(NTD)-Cerulean, which has been shown to interfere with the ability of PEX5(N-TPR) to disassemble oligomeric catalase tetramers (Freitas et al., 2011) (Figure 7A). First, we confirmed that the tagged proteins, PEX14(NTD)-EGFP and mCherry-PEX5(N-TPR), actually interact in living cells, and used mCherry-PEX5(TPR) as negative control (Figure 7B). The apparent interaction strength KD1^app^ obtained by bimolecular fitting was in the same range as PTS1 motifs (∼5.0E4 a. u.). Moreover, we demonstrated that overexpression of Cerulean-PTS1 (ACOX3) did not change the apparent interaction strength between PEX14(NTD) and PEX5(N-TPR), as overexpression of Cerulean-PTS1(ACOX1) did not affect the shape of the saturation curve (Figure 7C) and the comp:don dose response curve was flat (Figure 7D), a pattern, which was consistently found in three independent experiments (Figures 7E,F). This suggests that cargo-binding of PEX5(N-TPR) is not prerequisite for the interaction between PEX5(N-TPR) and PEX14(NTD).

Finally, we compared the modulatory effects of Cerulean-PTS1 (ACOX3) and PEX14(NTD)-Cerulean on the interaction between EGFP-PTS1 (ACOX3) and mCherry-PEX5(N-TPR) (Figures 7G–N). As expected, we found that increasing comp:don ratios of Cerulean-PTS1(ACOX3) progressively reduced the median DFRET values of subpopulations sharing the same acc:don ratios for mCherry-PEX5(N-TPR) as well as for mCherry-PEX5 (TPR) (Figure 7G, green). In contrast, the median DFRET values of mCherry-PEX5(N-TPR) were clearly reduced by PEX14(NTD)-Cerulean in a dose dependent, but not for mCherry-PEX5(TPR) although a comparison of the populations at a comp:don ratio between 1,6 and 2,4 showed a significant different due to the large number of cells although the effect size was very small (∼3.5%) (Figure 7G, blue). The decay curves due to Cerulean-PTS1 had similar shapes for PEX5(TPR) and PEX5(N-TPR) (Figure 7H, green), whereas PEX14(NTD) only caused a dose dependent decay in DFRET for mCherry-PEX5(N-TPR) (Figure 7H, blue). Interestingly, the decay curve caused by PEX14(NTD) seemed to have a different shape and appeared to reach a plateau above background level, which could reflect an allosteric inhibition, and was even clearer for higher acc:don ratios (Figure 7I), whereas no such pattern appeared when comparing the curve progression upon inhibition by Cerulean-PTS1 (Figure 7J). Similarly, the ascending phase of the saturation curve showed characteristic reductions in the slope upon overexpressing Cerulean-PTS1 for PEX5(TPR) and PEX5(N-TPR) (Figure 7K, green), whereas PEX14-Cerulean only reduced those of PEX5(N-TPR) (Figure 7K, blue). Importantly, the latter curve seemed to reach a lower DFRETmax plateau level, which is supported when inspecting curves based on a higher comp:don ratio (Figure 7L) but not for an inhibition by Cerulean-PTS1 (Figure 7M). Thus, overall, the curve progressions caused by PEX14(NTD) were obviously different from those caused by PTS1 as competitor and their shape resembled that of a non-competitive inhibition. These signs of another type of inhibition exerted by PEX14(NTD) on PEX5(N-TPR) were consistently found in three independent experiments (Figure 7N). However, as the presumable mode of inhibition is different for PEX14(NTD) the application of the fitting algorithm is not suitable and this allows only a qualitative description of the inhibitory effect. Altogether, these results not only verified the interaction between PEX5 and PEX14(NTD) in living cells, but also demonstrated that this interaction was not modulated by cargo proteins. On the contrary, PEX14(NTD) negatively modulated the interaction between PEX5(N-TPR) and its cargo, but the results provide evidence that the mode of inhibition is not a competitive one.

## 4 Discussion

The identification of PTS1 motifs, their ranking according to the affinity and the mode of recognition by the receptor PEX5 are long-standing research questions. However, investigating the interaction between full-length PTS1-carrying proteins and PEX5 has been a demanding task because this requires isolation and purification of proteins and the measurement of binding strengths by physical methods such as isothermal titration (Williams et al., 2011). In this manuscript, we describe a further development of our recently developed FRET-based method to study PEX5-PTS1 interactions in living cells (Hochreiter et al., 2020), which allows the measurement of the interaction strength between PEX5 and different full-length PTS1-containing cargo proteins. Although the coupling of FRET efficiency measurements and flow cytometry is a well-established method (Lim et al., 2022), our flowFRET approach unites an internal normalization procedure and mathematical analyses to estimate numerical values for apparent interaction strength and for the distance between donor and acceptor molecule. Here, we demonstrate that the overexpression of tagged competitor proteins, together with a suitable fitting algorithm, allows an efficient estimation of a measure for the interaction strength between the proteins. Using this approach, two traditional limitations of FRET measurements, namely the investigation of large proteins and low-affinity interactions can be effectively circumvented.

To verify the functionality of this approach, we demonstrate that the results of the prediction can depict characteristic properties of decay curves traditionally studied by binding assays, and the results of the fitting algorithms are in very good agreement with those obtained by bimolecular flowFRET measurements studying the same peptides directly. This method benefits from the internal normalization by concomitant measurement of the affinity of the acceptor to the donor (KD1) and the competitor (KD2), respectively. Thus, Log_10_(KD2^app^)/Log_10_(KD1^app^) are highly correlated under different conditions, such as differences in the composition of data sets with respect to the acc:don or comp:don ratio. This renders the results of the fitting algorithm more robust, especially when using the ratio Log_10_(KD2^app^)/Log_10_(KD1^app^) as output, and the results of experiments using the competition approach show lower normalized variability than bimolecular approaches. This competition method is especially well-suited to study the binding of low-affinity competitors such as the PTS1 of ACOX3-LK/SE or SCP2[AKL→AKV], which are difficult to study by bimolecular flowFRET experiments due to the low DFRET values of the entire sample. Moreover, we demonstrate that by this competition method, large proteins such as SCPx can be effectively studied with comparable variability to much smaller proteins such as SCP2. Finally, we provide the first evidence that this method might even be able to distinguish competitive modes of inhibition, for which the fitting algorithm has been developed, from other modes such as non-competitive or allosteric inhibition. Saturation (acc:don) and the decay (comp:don) curves caused by competitive inhibitors converge to the same plateau level or background level, whereas curves shaped by non-competitive mechanisms apparently present with different plateau levels. These differences simulate the curve progression for competitive and non-competitive inhibition of enzymes, but more detailed studies are needed and a mathematical solution for the fitting problem has to be developed.

**FIGURE 8:**
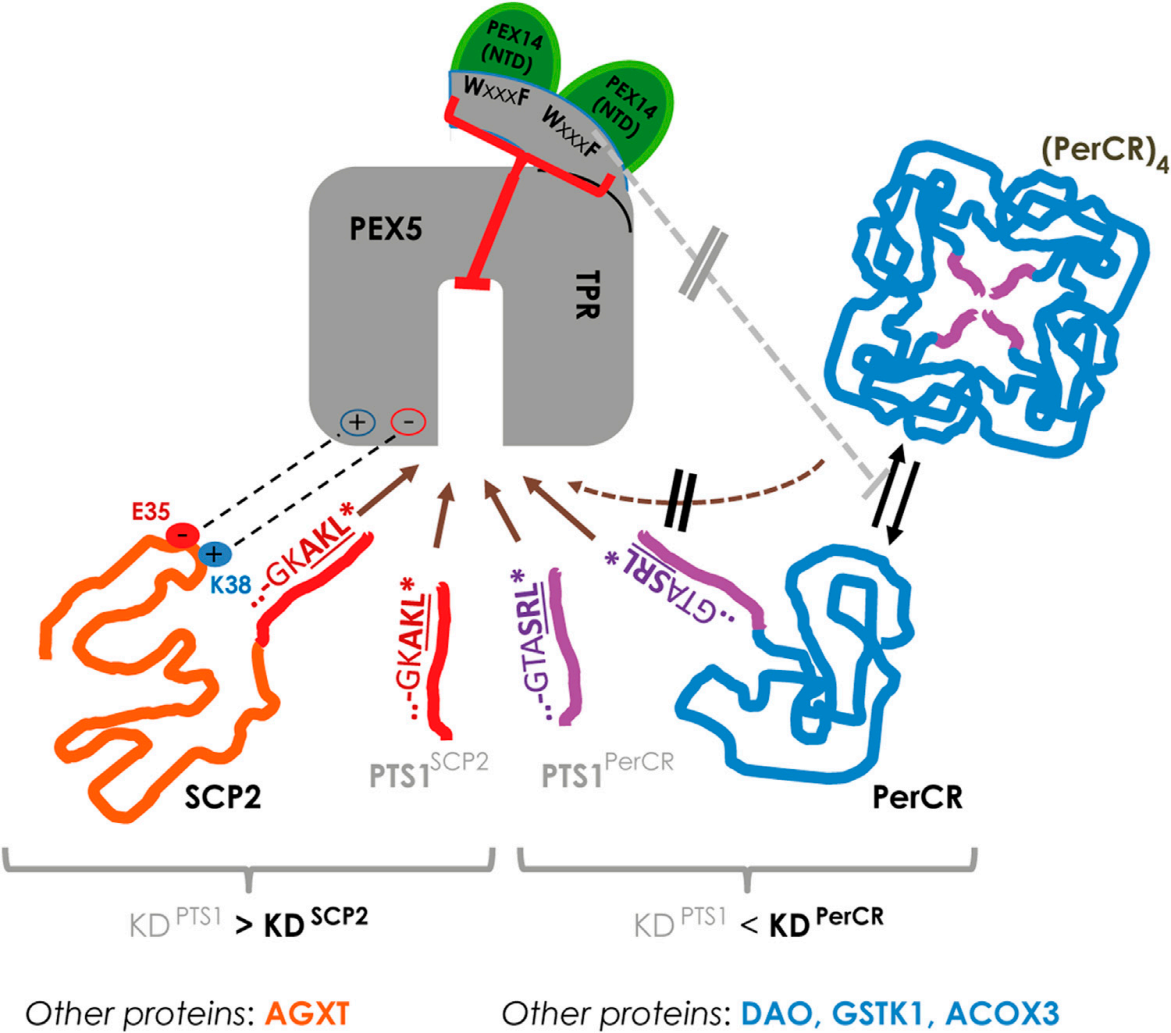
In living cells the interaction strength between PEX5 and its cargo proteins is modulated at various levels: *left*: in SCP2 the PTS1 binds PEX5 with lower affinity than the full-length protein due to an additional interaction domain (involving E35 and K38 of SCP2), which may also apply for the AGXT; *right*: in other proteins such as PerCR the interaction strength appears lower than the isolated PTS1, which is also observed for DAO, GSTK1 and ACOX3; in PerCR the PTS1 is inside the tetramer and thus not accessible to PEX5; thus, PerCR tetramers ((PerCR)_4_) cannot bind PEX5 (hatched brown arrow) reducing the apparent interaction strength, but this effect is not modulated by elongating the TPR domain of PEX5 (PEX5(N-TPR)) (grey line).

Applying flowFRET based competition to study the interaction between PEX5 and its cargo proteins, we were able to verify the surprisingly low affinity of the PTS1 of AGXT (about 13,5 µM (Ghosh and Berg, 2010)), which nonetheless mediates PEX5 binding and the import of full-length AGXT (Motley et al., 1995; Huber et al., 2005). Similar to AGXT, the affinity of the 12 amino acid long PTS1(SCP2) was markedly lower than that of SCP2 (summarized in Figure 8, left side), which agrees with previous results using the last 6 amino acids (Williams et al., 2011). Here, we retrace the higher affinity of SCP2 to the contribution of two charged residues in the N-terminus of SCP2 participating in the additional interphase between SCP2 and PEX5 and required to lift the affinity above that of the isolated PTS1(SCP2) peptide. However, we find that SCPx binds PEX5 with lower affinity than monomeric SCP2 and the apparent interaction strength of SCPx is more similar to that of isolated PTS1. As the C-terminal domain of SCP2 and SCPx is identical this difference is presumably caused by a shielding effect mediated by the N-terminal thiolase domain, which affects either directly the PTS1 or the additional N-terminal PEX5-binding domain of SCP2. Interestingly, the thiolase domain of the fish orthologoue (*D.rerio*) can occur as dimer (Kiema et al., 2019), which adds another level of complexity, because this dimerization also induces a conformational change in the thiolase domain. A similar difference between SCP2 and SCPx was observed when studying the effect of mutations directly in the PTS1, because we found that substituting a glycine of the PTS1 directly involved in PEX5-binding (Stanley et al., 2006) with the voluminous leucine (G139L) hardly affects the binding strength of SCPx to PEX5 but reduced that of SCP2 slightly. In contrast, substituting the C-terminal leucine by the equally bulky and hydrophobic valine (AKL→AKV) drastically reduced the affinity of SCP2 for PEX5, which can serve as explanation for the high abundance of leucine at the last position of naturally occurring tripeptides whereas valine is hardly found. This surprising result verifies in living cells for human PEX5 and a full-length protein a similar observation made by biophysical competition experiments using isolated plant PEX5 and PTS1-peptides (Skoulding et al., 2015). Surprisingly, the other full-length proteins investigated, such as *Hs*DAO, *Ss*PerCR, *Hs*ACOX3, and *Hs*GSTK1, presented with comparably low affinities compared to the PTS1 in isolation. However, only for SCP2 and AGXT a complex of cargo loaded PEX5 has been demonstrated by 3D-structures, whereas protein oligomerization and PEX5 binding might act competitively as has been described experimentally for human catalase (Freitas et al., 2011) or has been suggested based on steric restrictions in PerCR (Tanaka et al., 2008) (summarized in Figure 8, right side). Such competitive effects might contribute to our results, because in this system cargo proteins remain in the cytosol for a long time and thus might be prone to oligomerization. We exclude that an N-terminally extended variant of the PEX5 TPR-domain, which is able to bind PEX14 and was reported to disassemble catalase tetramers by a chaperone-like activity (Freitas et al., 2011) is sufficient to alleviate such competition effect, because the relative affinity of PEX5(TPR) and PEX5(N-TPR) to the investigated cargo proteins is similar. However, we cannot exclude the possibility that endogenous PEX14 might dissolve some of the cytosolic PEX5(N-TPR)-cargo complexes and thus hide a possible increase in affinity, although the high overexpression of donor and acceptor proteins is not favor of such model.

Finally, we provide evidence that the trimeric complex consisting of PEX5(N-TPR, aa268-602), PEX14(aa16–80) and SCP2, which has been reconstituted from isolated proteins (Shiozawa et al., 2009), also occurs in our system, because the interaction between PEX5(N-TPR) and PEX14(NTD) was well detectable, but not modulated by the presence of cargo proteins. Moreover, we also demonstrate that PEX14(NTD) reduces the affinity of human PEX5 (N-TPR) to a cargo protein, which had previously been shown for rat PEX14(NTD) in the context of inhibiting catalase tetramerization (Freitas et al., 2011). However, our results allow a quantifying description of the inhibitory effect, although the shape of the decay curve suggests a non-competitive mode of inhibition. Thus, these results further corroborate a PEX14-induced intraperoxisomal cargo release from PEX5 (Freitas et al., 2011), as a non-competitive mode of affinity modulation appears well suited for such mechanism (Figure 8).

Altogether, we introduce a novel method to study PPI in complexes involving large proteins by flowFRET and, by this means enlarged the toolbox for the peroxisome community to study the interaction between PEX5 and its full-length cargo proteins in more detail. Our experiments not only verified in living murine cells results obtained with isolated proteins but also provide the first description of a comparisons of different full-length PTS1-carrying proteins to PEX5.

## Data Availability

The raw data supporting the conclusions of this article will be made available by the authors, without undue reservation.

## Footnotes

1 Please note that in contrast to (Hochreiter, et al., 2020) we use here KD1^app^ as measure for interaction strength between donor and acceptor for which lower values indicate higher affinity.
