# Supplementary figures and images for: Studying the interaction between PEX5 and its full-length cargo proteins in living cells by a novel Försteŕs resonance energy transfer-based competition assay
Source: Front Cell Dev Biol. 2022 Nov 3;10:1026388. doi: 10.3389/fcell.2022.1026388 (PMC9669585; doi:10.3389/fcell.2022.1026388)

Suppl.Fig.1

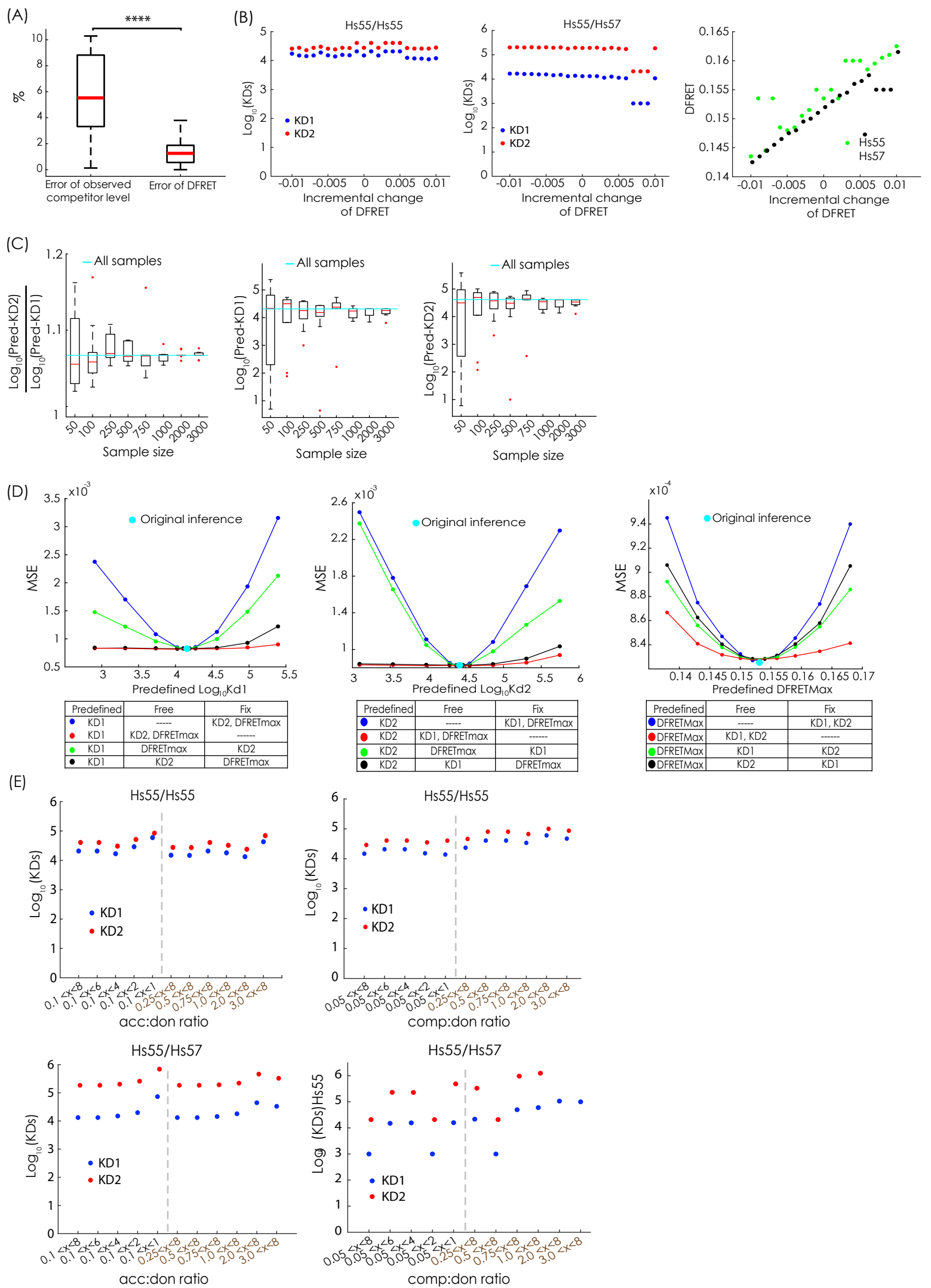

Supplement: Supplementary file 1 [file DataSheet2.PDF]

Suppl.Fig.3

(A) (B)

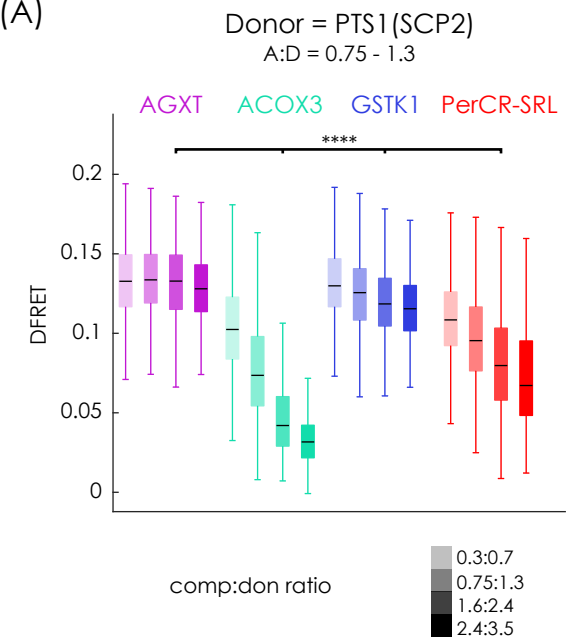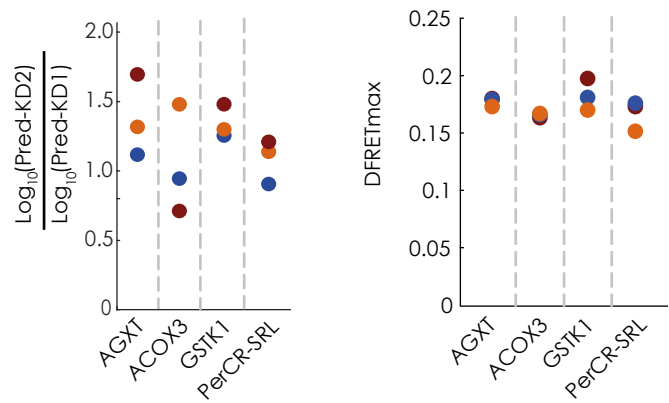

(C)

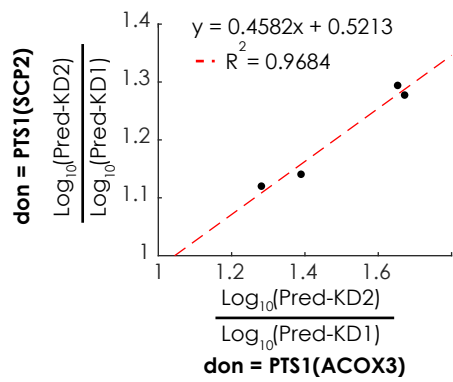

Supplement: Supplementary file 3 [file DataSheet4.PDF]

Suppl.Fig.2

(A)

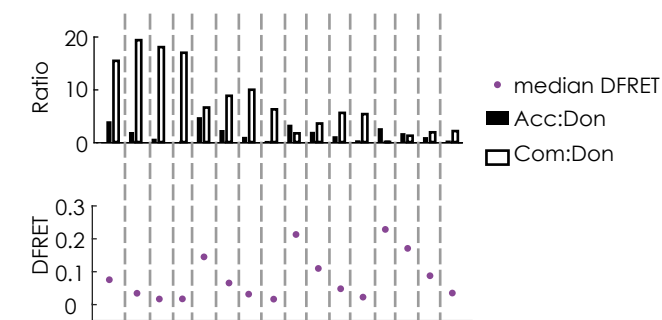

(B)

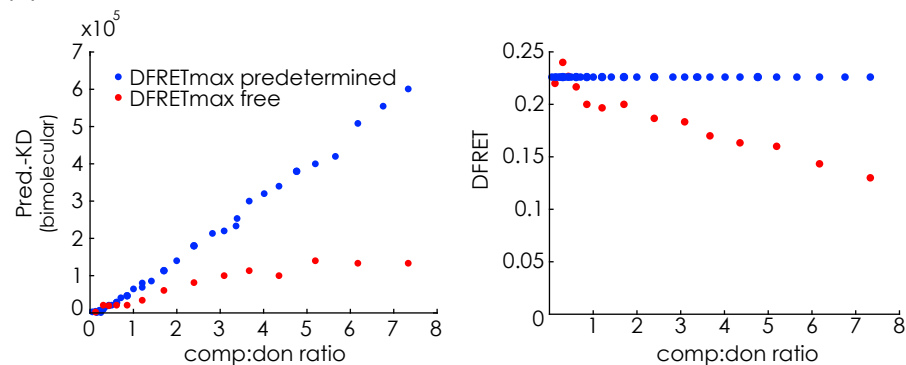

Supplement: Supplementary file 6 [file DataSheet3.PDF]
