# Supplementary material for: Studying the interaction between PEX5 and its full-length cargo proteins in living cells by a novel Försteŕs resonance energy transfer-based competition assay
Source: Front Cell Dev Biol. 2022 Nov 3;10:1026388. doi: 10.3389/fcell.2022.1026388 (PMC9669585; doi:10.3389/fcell.2022.1026388)
Supplement: Supplementary file 2 [file Table4.PDF]

**Core plasmids**

| | | | | |
|---|---|---|---|---|
| | EGFP-C1 | | | *Clontech* |
| | mCherry-C1 | | | *Clontech* |
| | Cerulean-C1 | | | *Clontech* |
| #1965 | mCherry-Cerulean | | | *Hochreiter, 2020* |
| #2003 | mCherry-EGFP | | | *Hochreiter, 2020* |

**Receptor**

| | | | | |
|---|---|---|---|---|
| #1932 | PEX5 | mCherry-PEX5 (TPR) | *H.sapiens* | *Hochreiter, 2020* |
| #2336 | PEX5 | mCherry-PEX5 (N-TPR) | *H.sapiens* | *this study* |
| #2413 | PEX14 | PEX14(N)-EGFP | *H.sapiens* | *this study* |
| #2424 | PEX14 | PEX14(N)-Cerulean | *H.sapiens* | *this study* |

**PTS1_Donor**

| | | | | |
|---|---|---|---|---|
| #2298 | SCP2 | EGFP-PTS1 (mSCP2) | *M.musculus* | *this study* |
| #2439 | AGXT | EGFP-PTS1 (AGXT) | *H.sapiens* | *this study* |
| #2382 | GSTK1 | EGFP-PTS1 GSTK1 | *H.sapiens* | *this study* |
| #2354 | DAO | EGFP-PTS1 (DAO) | *H.sapiens* | *Chong, 2019* |
| #2263 | ACOX3 | EGFP-PTS1 (ACOX3) | *H.sapiens* | *Hochreiter, 2020* |
| #2383 | PerCR-SRL | EGFP-PTS1 PerCR-SRL | *S.scrofa* | *this study* |

**Full-length Donor**

| | | | | |
|---|---|---|---|---|
| #566 | SCPX | EGFP-SCPX | *M.musculus* | *this study* |
| #2302 | SCP2 | EGFP-SCP2 | *M.musculus* | *this study* |
| #2401 | EGFP-AGXT | EGFP-AGXT | *H.sapiens* | *Fodor, 2015* |
| #2449 | SCP2-AKV | EGFP-SCP2-AKV | *M.musculus* | *this study* |
| #2450 | SCP2-G139L | EGFP-SCP2-G139L | *M.musculus* | *this study* |
| #2511 | SCP2-E35K/K38E | EGFP-SCP2 (E35K/K38E) | *M.musculus* | *this study* |

**PTS1 Competitor**

| | | | | |
|---|---|---|---|---|
| #2343 | SCP2 | Cerulean-PTS1-SCP2 | *M.musculus* | *this study* |
| #2274 | PTS1 (Hs55) | Cerulean-PTS1 (Hs55) | *2H-peptide* | *Hochreiter, 2020* |
| #2285 | PTS1 (Hs57) | Cerulean-PTS1 (Hs57) | *2H-peptide* | *Hochreiter, 2020* |
| #2339 | ACOX3 | Cerulean-PTS1 (ACOX3) | *H.sapiens* | *this study* |
| #2341 | ACOX3 LK/SE | Cerulean-PTS1 (ACOX3 LK/SE) | *H.sapiens* | *this study* |
| #2369 | ACOX3 K-1E | Cerulean-PTS1 (ACOX3 K-1E) | *H.sapiens* | *this study* |

**Full-length Competitor**

| | | | | |
|---|---|---|---|---|
| #2344 | SCP2 | Cerulean-SCP2 | *M.musculus* | *this study* |
| #2389 | SCP2 (AKV) | Cerulean-SCP2 (AKV) | *M.musculus* | *this study* |
| #2390 | SCP2 (G139L) | Cerulean-SCP2 (G139L) | *M.musculus* | *this study* |
| #2342 | SCPx | Cerulean-SCPx | *M.musculus* | *this study* |
| #2387 | SCPx (AKV) | Cerulean-SCPx (AKV) | *M.musculus* | *this study* |
| #2388 | SCPx (G139L) | Cerulean-SCPx (G139L) | *M.musculus* | *this study* |
| #2445 | AGXT | Cerulean-AGXT | *H.sapiens* | *this study* |
| #2452 | GSTK1 | Cerulean-GSTK1 | *H.sapiens* | *this study* |
| #2467 | DAO | Cerulean-DAO | *H.sapiens* | *this study* |
| #2454 | ACOX3 | Cerulean-ACOX3 | *H.sapiens* | *this study* |
| #2453 | PerCR-SRL | Cerulean-PerCR-SRL | *S.scrofa* | *this study* |

**PEX14**

| | | | | |
|---|---|---|---|---|
| Oli_1529 | CTCGAGatggcgtcgtcggagcaggc | fw | PEX14 N-terminus | XhoI |
| Oli_2799 | ggatccCggtagctgggcccagg | rev | PEX14 | BamHI |

**Full-length proteins**

| | | | | |
|---|---|---|---|---|
| Oli_901 | CGCTGCTCGAGCCATG | fw | mSCPx | XhoI |
| Oli_902 | CCAAAGGAATTCCTCACAGCTTA | rev | mSCPx | EcoRI |
| Oli_2615 | ctcgagccatgggttttcccgaagctgccagc | fw | SCP2 START | XhoI |
| Oli_2736 | GAATTCCTCACACCTTAGCTTTGCCCGGCTG | rev | SCPx-AKV | EcoRI |
| Oli_2737 | GAATTCCTCACAGCTTAGCTTTGAGCGGCTGAAG | rev | SCPx-G139L | EcoRI |
| Oli_2719 | AGATCTatgcgggcggcggggcagt | fw | SsPerCR_PCR | BglII |
| Oli_2720 | GTCGACCCTGGTGGATGCTCTCCAG | rev | SsPerCR_PCR | SalI |
| Oli_2738 | AGATCTATGGCCAGCACCGGGGTG | fw | SsPerCR_PCR | BglII |
| Oli_3002 | tcgtctttaagAagattgagGagaaActtgaagagga | fw | mSCP2 (E35K/K38E) | destroys HindIII |
| Oli_3003 | tcctcttcaagTttctCctcaatctTcttaaagacga | rev | mSCP2 (E35K/K38E) | destroys HindIII |

**PTS peptides**

| | | | | |
|---|---|---|---|---|
| Oli_2613 | GATCTAcagaaccttcagcttcagccgggcaaagctaagctgtgaA | fw | PTS1(SCP2) | BglII |
| Oli_2614 | AGCTTtcacagcttagctttgcccggctgaagctgaaggttctgTA | rev | PTS1(SCP2) | HindIII |
| Oli_2910 | GATCTAagggcggccctgcagcactgccccaagaagaagctgtgaA | fw | PTS1_AGXT | BglII |
| Oli_2911 | AGCTTtcacagcttcttcttggggcagtgctgcagggccgccctTA | rev | PTS1_AGXT | HindIII |
| Oli_2713 | GATCTAAtgggccctatacctccagccgtgaatgccagactttgaA | fw | PTS1_GSTK1Hs | BglII |
| Oli_2714 | AGCTTtcaaagtctggcattcacggctggaggtatagggcccatTA | rev | PTS1_GSTK1Hs | HindIII |
| Oli_2715 | GATCTAACAGTGGTAGTGGGTGGAGGGACCGCATCCCGCCTCtgaA | fw | PTS1_SsPerCR | BglII |
| Oli_2716 | AGCTTtcaGAGGCGGGATGCGGTCCCTCCACCCACTACCACTGTTA | rev | PTS1_SsPerCR | HindIII |
