# Supplementary material for: Studying the interaction between PEX5 and its full-length cargo proteins in living cells by a novel Försteŕs resonance energy transfer-based competition assay
Source: Front Cell Dev Biol. 2022 Nov 3;10:1026388. doi: 10.3389/fcell.2022.1026388 (PMC9669585; doi:10.3389/fcell.2022.1026388)
Supplement: Supplementary file 5 [file Table2.PDF]

Results of prediction

Acceptor	Donor	Competitor/Modulator	n (exp.)	Descritptive Statistics: log10 (KD1) Mean	log10 (KD1) STABW	log10(KD2)/log10(KD1) Mean	log10(KD2)/log10(KD1) STABW	DFRETmax Mean	DFRETmax STABW	Specific research questions: Boots-trapping	Kolmogorov-Smirnov	Paired t-Test	p-value	p-value
**Fig.2**													log10(KD)	DFRETmax
**F (bimolecular)**														
PEX5(TPR)	**PTS1 (SCP2)**		9	4,4747	0,3738			0,1778	0,0169			**PTS1 - SCP2**	**0,001537**	0,002219
PEX5(TPR)	**SCP2**		9	3,7641	0,6942			0,1620	0,0144			**SCP2 - SCPx**	**0,220729**	0,050357
PEX5(TPR)	**SCPx**		4	4,9275	1,4824			0,1103	0,0434					
**J (bimolecular)**														
PEX5(TPR)	**SCP2**		4	3,2430	0,2909			0,1677	0,0150				log10(KD)	
PEX5(TPR)	**SCP2 (AKV)**		4	4,4205	0,7370			0,1663	0,0293			**SCP2 - AKV**	**0,034106**	
PEX5(TPR)	**SCP2 (G139P)**		4	3,6413	0,4920			0,1515	0,0085			**SCP2 - G139L**	0,083741	
**N (bimolecular)**													log10(KD)	DFRETmax
PEX5(TPR)	**PTS1 (AGXT)**		5	5,5759	1,0299			0,2012	0,1094			**PTS1(AGXT) - AGXT**	0,072347	0,232384
PEX5(TPR)	**AGXT**		5	4,8759	0,3204			0,1478	0,0354					
**Fig.3**														
**H (bimolecular)**											**Kol.Smir.**			
PEX5(TPR)	**PTS1(ACOX3)**		14	3,8337	0,5669			0,1802	0,0196		not sign.			
PEX5(TPR)	PTS1(ACOX3)	**PTS1(ACOX3)**	11			1,1061	0,0814	0,1814	0,0168		not sign.			
**Fig.4**														
**F (competition)**										**Boots-trapping**				
PEX5(TPR)	PTS1(ACOX3)	**PTS1 (ACOX3)**	3			1,1067	0,0486	0,1909	0,0159	**p = 0.000076**				
PEX5(TPR)	PTS1(ACOX3)	**PTS1 (ACOX3 K-1E)**	3			1,2667	0,0897	0,2014	0,0406					
PEX5(TPR)	PTS1(ACOX3)	**PTS1 (ACOX3 LK/SE)**	3			1,3767	0,1115	0,2033	0,0408					
PEX5(TPR)	PTS1(ACOX3)	**PTS1 (Hs55)**	3			1,1934	0,0470	0,1986	0,0356					
PEX5(TPR)	PTS1(ACOX3)	**PTS1 (Hs57)**	3			1,2960	0,1174	0,2071	0,0509					
**K (competition)**													log10(KD2)/log10(KD1)	
PEX5(TPR)	PTS1(SCP2)	**PTS1(SCP2)**	5			1,0408	0,0086	0,2005	0,0276			**PTS1(SCP2) - SCP2**	**0,000261**	
PEX5(TPR)	PTS1(SCP2)	**SCP2**	5			0,8545	0,0326	0,1961	0,0308			**SCP2 - SCPx**	**0,007986**	
PEX5(TPR)	PTS1(SCP2)	**SCPx**	5			1,0436	0,0597	0,1866	0,0119			**PTS1(SCP2) - SCPx**	0,92533	
**O (competition)**													log10(KD2)/log10(KD1)	
PEX5(TPR)	PTS1(SCP2)	**SCP2**	4			0,8528	0,0373	0,1990	0,0347			**SCP2 - SCP2(AKV)**	**0,001389**	
PEX5(TPR)	PTS1(SCP2)	**SCP2 (AKV)**	4			1,1752	0,0358	0,2063	0,0296			**SCP2 - SCP2(G139L)**	0,055223	
PEX5(TPR)	PTS1(SCP2)	**SCP2 (G139L)**	4			0,9156	0,0505	0,2051	0,0362					
PEX5(TPR)	PTS1(SCP2)	**SCPx**	4			1,0545	0,0629	0,1855	0,0134			**SCPx - SCPx(AKV)**	**3,68E-05**	
PEX5(TPR)	PTS1(SCP2)	**SCPx (AKV)**	4			1,1688	0,0637	0,1922	0,0207			**SCPx - SCPx(G139L)**	0,996597	

PEX5(TPR)	PTS1(SCP2)	**SCPx (G139L)**	4			1,0544	0,0816	0,1945	0,0244

Fig.5

D (competition)

PEX5(TPR)	PTS1(ACOX3)	**AGXT**	4			1,6529	0,1498	0,1722	0,0092
PEX5(TPR)	PTS1(ACOX3)	**ACOX3**	4			1,2812	0,1150	0,1754	0,0047
PEX5(TPR)	PTS1(ACOX3)	**DAO**	4			1,4943	0,1400	0,1753	0,0054
PEX5(TPR)	PTS1(ACOX3)	**GSTK1**	4			1,6719	0,1007	0,1748	0,0047
PEX5(TPR)	PTS1(ACOX3)	**PerCR-SRL**	4			1,3893	0,1046	0,1740	0,0027

Boots-trapping
p = 3.72e-4

G (bimolecular)

PEX5(TPR)	**PTS1 (SCP2)**		3	4,3916	0,1898			0,1800	0,0020
PEX5(TPR)	**PTS1 (AGXT)**		3	5,8787	0,7610			0,1700	0,0849
PEX5(TPR)	**PTS1 (ACOX3)**		3	3,3979	0,6892			0,1773	0,0050
PEX5(TPR)	**PTS1 (DAO)**		3	5,0725	0,0628			0,1556	0,0065
PEX5(TPR)	**PTS1 (GSTK1)**		3	4,7235	0,0996			0,1676	0,0051
PEX5(TPR)	**PTS1 (PerCR-SRL)**		3	5,0157	0,0669			0,1800	0,0087

Boots-trapping
p = 2e-6

Fig.6

C (bimolecular)

**PEX5 (TPR)**	PTS1 (ACOX3)		4	3,8364	0,1725			0,1685	0,0030
**PEX5 (N-TPR)**	PTS1 (ACOX3)		4	3,9563	0,1918			0,1607	0,0078
**PEX5 (TPR)**	PTS1 (SCP2)		3	4,3084	0,2317			0,1698	0,0144
**PEX5 (N-TPR)**	PTS1 (SCP2)		3	4,4992	0,2354			0,1507	0,0162

	log10(KD)	DFRETmax
**TPR - (N-TPR)**	0,338971	0,149158
**TPR - (N-TPR)**	**0,000435**	**0,006958**

F (bimolecular)

PEX5 (N-TPR)	**PTS1**		3	4,7235	0,0996			0,1616	0,0014
PEX5 (N-TPR)	**SCP2**		3	3,2377	0,1916			0,1320	0,0053
PEX5 (N-TPR)	**SCP2 (E35K/K38E)**		3	4,3336	0,0269			0,1440	0,0035

	log10(KD)	DFRETmax
**PTS1(SCP2) - SCP2**	**0,00226**	**0,005772**
**SCP2 - SCP2(E35K/K38E)**	**0,012517**	

J (competition)

PEX5 (N-TPR)	PTS1(ACOX3)	**AGXT**	3			1,6468	0,1258	0,1658	0,0008
PEX5 (N-TPR)	PTS1(ACOX3)	**ACOX3**	3			1,1857	0,1180	0,1649	0,3994
PEX5 (N-TPR)	PTS1(ACOX3)	**DAO**	3			1,5577	0,1886	0,1677	0,3002
PEX5 (N-TPR)	PTS1(ACOX3)	**GSTK1**	3			1,6652	0,1714	0,1655	0,1476
PEX5 (N-TPR)	PTS1(ACOX3)	**PerCR-SRL**	3			1,3205	0,0676	0,1612	0,1152

Boots-trapping
p = 8.04e-4

Fig.7

B (bimolecular)

PEX5 (N-TPR)	**Pex14(N)**		3	4,6414	0,2805			0,2196	0,0156

C&D (bimolecular)

PEX5 (N-TPR)	Pex14(N)	**Cerulean**	3	4,5985	0,0173			0,2329	0,0094
PEX5 (N-TPR)	Pex14(N)	**PTS1 (ACOX3)**	3	4,5017	0,1598			0,2258	0,0049

	log10(KD)
**Cerulean - PTS1(ACOX3)**	0,360461

N (bimolecular)

PEX5(TPR)	PTS1(ACOX3)	**Cerulean**	3	3,5585	0,5829	0,1762	0,0050
PEX5(TPR)	PTS1(ACOX3)	**PEX14(N)**	3	3,5297	0,6960	0,1764	0,0034
PEX5(TPR)	PTS1(ACOX3)	**PTS1 (ACOX3)**	3	4,7639	0,1774	0,1376	0,0107
PEX5 (N-TPR)	PTS1(ACOX3)	**Cerulean**	3	3,6301	0,5241	0,1700	0,0040
PEX5 (N-TPR)	PTS1(ACOX3)	**PEX14(N)**	3	2,7730	0,5165	0,1031	0,0090
PEX5 (N-TPR)	PTS1(ACOX3)	**PTS1 (ACOX3)**	3	4,5525	0,2120	0,1196	0,0032

		log10(KD)	DFRETmax
**TPR**	**Ceru.-PEX14(N)**	0,866323	0,90241
	**Ceru.-PTS1(ACOX3)**	**0,039575**	**0,013495**
**N-TPR**	**Ceru.-PEX14(N)**	**0,0121**	**0,004419**
	**Ceru.-PTS1(ACOX3)**	0,077505	**0,005791**
**PEX14**	**TPR - (N-TPR)**	0,105827	**0,002005**
**PTS1(ACOX3)**	**TPR - (N-TPR)**	0,124782	0,054231
