# Supplementary material for: Studying the interaction between PEX5 and its full-length cargo proteins in living cells by a novel Försteŕs resonance energy transfer-based competition assay
Source: Front Cell Dev Biol. 2022 Nov 3;10:1026388. doi: 10.3389/fcell.2022.1026388 (PMC9669585; doi:10.3389/fcell.2022.1026388)
Supplement: Supplementary file 7 [file DataSheet1.PDF]

Supplementary figures & tables

Suppl.Fig.1:

(A) **Estimate the error due to misjudgement of Cerulean-mCherry**: Although FRET efficiency between Cerulean and mCherry fluorescent proteins has been observed, the presence of Cerulean does not directly affect the measurement of the FRET efficiency between EGFP and mCherry, because Cerulean is excited as a shorter wave length, but facilitates a misjudgment of the level of Cerulean. On the one hand the normalization curve serving to convert the intensity in the Cerulean channel into that of the mCherry channel has an incorrect slope and on the other hand the cellular intensity of Cerulean is underestimated due to energy transfer to mCherry within the complex between mCherry-PEX5(TPR) and Cerulean-PTS1. Whereas the first factor has been taken care by recalculating the slope of the normalization curve (Hochreiter, 2020), the extent of misjudgment of Cerulean due to intracellular FRET was unclear. Based on the results obtained by the mCherry-Cerulean fusion protein, the DFRETmax value for the corresponding protein complex (mCherry-PEX5(TPR) & Cerulean-PTS1) was estimated to be about 10,33% for the PTS1-PEX5(TPR) complex, which was consistently delineated from a saturation curve and by recalculation from the transfer efficiency within the fusion protein presuming the same change in efficiency when changing from the fusion protein to the situation in the PTS1-PEX5(TPR) complex. To better understand the consequences of a misjudgment of the level of the Cerulean level on the estimation of FRET-relevant acc.don complexes, we used of the values of the inferred obtained by the fitting algorithm to the data set Hs55/Hs55 and first calculated the amount of acc.comp complexes within each cell and by multiplying this value with the estimated DFRETmax for Cerulean-mCherry transfer in the PTS1-PEX5(TPR) complex (0,1033) the putative loss of Cerulean by $FRET^{Ceru=>mCherry}$ was estimated. Adjusting the Cerulean level ($Cerulearn^{corr}$) allowed us then to calculate the putative change in the expected change in the amount of FRET-relevant acc.don complexes. The average error size of the Cerulean intensity value was around 5,87 +/- 2,89% and using the corrected values for Cerulean the predicted changes in acc.don complexes was about 1,27 +/- 0,851%. When we used these corrected values for the Cerulean channel ($Ceru^{corr}$) and applied the fitting algorithm to the modified data set we obtained very similar values ($KD1^{app}$ = 13534,5 instead of 14076; $KD2^{app}$ = 26731 instead of 26258, and DFRETmax = 0,154 instead of 0,153). This demonstrated that even for competitors with high affinity and high DFRETmax due to a small size the error caused by an incorrect Cerulean-estimation is negligible, although this effect further decreases upon increasing the size of the competitor (lower $FRET^{Ceru=>mCherry}$) and for low affinity competitors (less competitor is bound). Statistics: Wilcoxon signed rank test ($p < 1e-50$)

(B) **Effect of misjudgment of the DFRET background value:** To verify the robustness of our fitting result from a minor misjudgment of the background level of the FRET channel we took advantage of data sets obtained by the competition flowFRET experiment using donor and competitor sharing the same PTS1-peptide (Hs55/Hs55) (left) or a much weaker competitor (Hs55/Hs57) (middle) and incrementally increased or decreased the actual DFRET value of all data points and resubmitted the modified data sets to the fitting algorithm. We found that the predicted values for $Log_{10}(KD1^{app})$ (blue) and $Log_{10}(KD2^{app})$ (red) were rather robust against changes in the background level and for those samples, in which one value changed the other did as well. This suggested that the ratio of $Log_{10}(KD2^{app})/Log_{10}(KD1^{app})$ is more

reliable than the individual predicted values. Moreover, the artificial change in the DFRET value of the data points was well-reflected by corresponding changes in the predicted DFRETmax (right) although the inference from data sets including the low-affinity competitor (Hs57) appeared more affected (green).

(C) **Reliability of the inferred values depends on the sample size:** To estimate the reliability of the computational extraction of values for $KD1^{app}$ and $KD2^{app}$ and DFRETmax, we randomly collected ten subsamples each of different sizes (n = 50, 100, 250, 500, 750, 1000, 2000 and 3000) and subjected these data sets to the fitting algorithm. We found that from a size of 250 the median of the different $Log_{10}(KD2^{app})/Log_{10}(KD1^{app})$ (left) values was reliable, but until a sample size of 750 occasional outliers rendered the individual estimations not reliable. This was also observed for the independent prediction of Log_{10}(Pred-$KD1^{app}$) (middle) and Log_{10}(Pred-$KD2^{app}$) (right).

(D) **Compensatory adaptation of the fitting algorithm upon forced deviation from the inferred values:** As the mathematical solution of the first derivatives of the complex equation was not accessible to us, we determined the relative sensitivity of the result to misjudgements of KD1app, KD2app or DFRETmax using the mean square error (MSE) as criterion. Based on the data set obtained by the competition flowFRET experiment using donor and competitor sharing the same PTS1-peptide (Hs55/Hs55) the inferred values for $KD1^{app}$ and $KD2^{app}$ and DFRETmax were used. Next, either $KD1^{app}$ (left), $KD2^{app}$ (middle) or DFRETmax (right) were incrementally changed and predefined (+ fold: 1.1; 1.33; 2.6; 6.76; 17.56; -fold: 0.91; 0.75; 0.39; 0.15; 0.057) and the change in the mean error per point (MSE) was plotted against the change in the value when either the two other parameters were kept constant (blue), or when both other parameter were free to adapt (red) or when one of the other parameters was kept constant, whereas the other was free (green, black). The same change in one parameter was also used to investigate the efficiency of a counterbalancing adaptation of one of the other parameters. In this case, the change of one parameter is expected to be accompanied by a change in another parameter to minimize the MSE (*left: $KD1^{app}$; middle: $KD2^{app}$ and right DFRETmax*). In summary, changes in one of the inferred values for $KD1^{app}$ or $KD2^{app}$ were accompanied by a marked increase in the mean error, whereas an adaptive change in the other KD^{app} was able to compensate this effect over large ranges corroborating the interdependence of the two values. In contrast, changes in the value of DFRETmax had marked effects on the mean error without effective compensatory adaptations of $KD1^{app}$ or $KD2^{app}$ (right). This is in good agreement with the shape of the inhibitor mediated decay curve, in which the slope it directly related to the ratio of $KD2^{app}/KD1^{app}$. The original MSE value at the 0-level is indicated as blue dot.

(E) **Restricting data set for acc:don and comp:don ranges**: To further corroborate this observation and to test the relevance of the composition of the data set, we generate subsets of Hs55/Hs55 and Hs55/Hs57 by defining ranges of acc:don or comp:don ratios and independently extracted $KD1^{app}$, $KD2^{app}$ and DFRETmax for these subsets. We found that restricting the data set can markedly affect the values predicted for each of the parameters, but surprisingly, when plotting $Log_{10}(KD1^{app})$ against $Log_{10}(KD2^{app})$ the different values appeared highly correlated and the ratio of $Log_{10}(KD2^{app})/Log_{10}(KD1^{app})$ appeared more robust than individual measurements of $Log_{10}(KD1^{app})$ or $Log_{10}(KD2^{app})$.

Suppl.Fig.2:

(A) **Median DFRET values reflect the composition the data set:** A series of independent data sets were generated by flowFRET competition experiments using EGFP-PTS1(ACOX3) as donor and Cerulean-PTS1(ACOX3) as acceptor, in which each sample originated from an independent transformation using different ratios of expression plasmids encoding acceptor, donor and competitor protein (*cf*. Fig.2F). When plotting the median DFRET values of these 16 independent transformations and relating these values with the acc:don ratios (black rectangle) or the comp:don ratios (white rectangle) we found that high median DFRET values are found at high acc:don ratios, whereas low median DFRET values were found in the presence of high comp:don ratios.

(B) **Increasing levels of competitor reduces the $KD1^{app}$ obtained by inference using the bimolecular equation**: To test the internal consistency of the data set described in Fig.2F and (A) we generated a large data set by uniting the 16 data sets and then divided it into subpopulations sharing the same comp:don ratio. Each data set is subjected to the bimolecular fitting algorithm independently either allowing free adjustment of $KD1^{app}$ and DFRETmax (red) or upon fixation of DFRETmax (blue) to the value obtained by fitting the entire data set. We found that increasing comp:don ratios are accompanied with increasing $KD1^{app}$(left), but without fixation the adaptation of DFRETmax (right) overrules the change in $KD1^{app}$.

Suppl.Fig.3:

FlowFRET competition experiment of full-length PTS1-carrying proteins using EGFP-PTS1(SCP2) as donor: Competition flowFRET experiment using EGFP-PTS1 (SCP2) as donor, mCherry-PEX5(TPR) as acceptor, and different Cerulean fusion proteins as competitor (Cerulean-AGXT, Cerulean -AGXT, Cerulean -GSTK1, Cerulean -PerCR-SRL). $pex5^{-/-}$ MEF expressing mCherry-PEX5(TPR) as acceptor and EGFP-PTS1(SCP2) as donor various donor and various competitor molecules were analyzed by flow cytometry to obtain a large number of cells; competitor proteins: Cerulean-tagged full length proteins of hAGXT, hACOX3, hGSTK1, pig PerCR;

(A) the median DFRET values of subpopulations sharing an acc:don ratio of 0,75<x<1,3 and different competitor to donor ratios are depicted;
(B) the results of the fitting (Pred.-KD1, Pred.-KD2 and DFRETmax) are plotted as ratio of Log_{10}(Pred.-KD2)/Log_{10} (Pred.-KD1) or as DFRETmax;
(C) plotting the Log_{10}(Pred.-KD2)/Log_{10} (Pred.-KD1) of experiments using either EGFP-PTS1(ACOX3) as donor (*Cf*.Fig.4D) (x-axis) and EGFP-PTS2(SCP2) as donor (y-axis) reveal a correlation between the resulting ratios. The shift to lower values reflects the lower affinity and thus the higher Log_{10}(Pred.-KD1) of PTS1(SCP2) in the denominator.

Suppl.Table 1: Overview of descriptive and comparative statistics of the experimental results.

Suppl.Table 2: Overview of descriptive and comparative statistics of the results of computational inferrence.

Suppl.Table 3: Overview of the plasmids and oligonucleotides used in this study.
